# Carotenoids from Marine Organisms: Biological Functions and Industrial Applications

**DOI:** 10.3390/antiox6040096

**Published:** 2017-11-23

**Authors:** Christian Galasso, Cinzia Corinaldesi, Clementina Sansone

**Affiliations:** 1 Stazione Zoologica Anton Dohrn, Villa Comunale, 80121 Naples, Italy; christian.galasso@szn.it; 2 Department of Sciences and Engineering of Materials, Environment and Urbanistics, Università Politecnica delle Marche, 60121 Ancona, Italy

**Keywords:** marine carotenoids, biological functions, antioxidants, industrial applications, antioxidant

## Abstract

As is the case for terrestrial organisms, carotenoids represent the most common group of pigments in marine environments. They are generally biosynthesized by all autotrophic marine organisms, such as bacteria and archaea, algae and fungi. Some heterotrophic organisms also contain carotenoids probably accumulated from food or partly modified through metabolic reactions. These natural pigments are divided into two chemical classes: carotenes (such as lycopene and α- and β-carotene) that are composed of hydrogen and carbon; xanthophylls (such as astaxanthin, fucoxanthin and lutein), which are constituted by hydrogen, carbon and oxygen. Carotenoids, as antioxidant compounds, assume a key role in the protection of cells. In fact, quenching of singlet oxygen, light capture and photosynthesis protection are the most relevant biological functions of carotenoids. The present review aims at describing (i) the biological functions of carotenoids and their benefits for human health, (ii) the most common carotenoids from marine organisms and (iii) carotenoids having large success in pharmaceutical, nutraceutical and cosmeceutical industries, highlighting the scientific progress in marine species cultivation for natural pigments production.

## 1. Introduction

Since the first structural elucidation of β-carotene by the two scientists Kuhn and Karrer in 1930, about 750 naturally occurring carotenoids have been reported. Among these bioactive compounds, more than 250 are of marine origin and show an interesting structural diversity, such as in the case of allenic carotenoids (e.g., fucoxanthin) and all acetylenic carotenoids (e.g., tedaniaxanthin and alloxanthin) originated from marine algae and animals [1,2]. Marine carotenoids exert strong antioxidant, repairing, antiproliferative and antiinflammatory effects and can be used either as skin photo-protection to inhibit adverse effects of solar UV radiation or as nutraceutical/cosmeceutical ingredients to prevent oxidative stress-related diseases [3,4,5]. Except for autotrophic marine organisms, non-photosynthetic marine organisms are not able to synthesize carotenoids de novo. Therefore, marine animals can either directly accumulate carotenoids from food or partly modify them through metabolic mechanisms. The main metabolic conversions of carotenoids found in animals are translation and oxidative cleavage of double bonds, oxidation, reduction and cleavage of epoxy bonds [6,7,8,9,10]. Among heterotrophic organisms, bacteria, archaea, marine fungi (especially pigmented yeasts) and thraustochytrids (generally defined as fungi-like protists) are a relevant source of carotenoids [11]. Currently, the most common carotenoids that are commercially available are industrially produced by chemical synthesis which increases production costs and waste materials production with potential negative effects on the environment [12]. In the last decade, the increasing consumer concern for human and environmental health has driven the growth of the market demand for natural carotenoids-based products. Therefore, the biotechnological regarding the production of natural carotenoids from marine organisms (especially microalgae and seaweeds) has considerably increased, because they can offer several advantages in terms of costs, times and yields when compared to terrestrial plants or synthetic products. In addition, the industrial production of carotenoids extracted from terrestrial plants is generally dependent on the seasons and geographic areas, which cannot always be standardized. The present review describes progress in the scientific research of carotenoids extracted from marine organisms over the last decades highlighting sources, biological roles and industrial applications. The extraction of carotenoids from marine organisms, especially microorganisms (both autotrophic and heterotrophic such as microalgae, bacteria, archaea, fungi and fungi-like protists) can be performed by controlling and optimizing their growth conditions and using low-cost and fast approaches, thus representing an economically valid and biosustainable alternative production of carotenoids.

## 2. Biological Functions of Carotenoids

### 2.1. Role of Carotenoids in the Prooxidant-Antioxidant Balance

Reactive species are foremost known for their harmful effects and have a wide range of physiological functions. In particular, lipids, nucleic acids (RNA and DNA) and proteins represent the main targets of reactive oxygen species (ROS), reactive nitrogen species (RNS) and reactive sulphur species (RSS) [13,14]. In addition, radical species can have a protective effect in the inflammatory processes; can be involved in mitochondrial free radical production as part of the Tumor Necrosis Factor (TNF) signaling during apoptosis and in the protein phosphorylation mechanisms [15,16,17]. The large class of antioxidants, which are defined as any chemical substance that is able to delay, reduce, modify, prevent or remove oxidative damage, represents the chemical counterpart of free radical species protecting target structures or molecules from oxidative injuries [18]. The antioxidant machinery developed by humans, although highly efficient, cannot operate only with endogenous compounds, but depends on a plethora of exogenous antioxidants that humans obtain from the daily diet.

Natural carotenoids exert their functions in different ways and at different levels. In fact, they can: (i) act as quenchers of singlet molecular oxygen; (ii) convert hydroperoxides into more stable compounds; (iii) can prevent formation of free radicals through the block of free radical oxidation reactions and inhibition of the autoxidation chain reaction; and (iv) convert iron and copper derivatives (metal prooxidants) into harmless molecules, acting as metal chelators. Due to this double face, participation to key physiological mechanisms and their potential harmful effects, the balance between prooxidant and antioxidant molecules in the human body is controlled through a fine regulation system. The oxidative stress arises when the balance between formation of free radicals and their neutralization through the antioxidant machinery tends to the overproduction of reactive species, with negative consequences for cells and extracellular structures [19]. For this reason, deregulation of redox balance for extended periods is responsible of the increased incidence of cancers, cardiovascular diseases, photosensitivity disorders, diabetes, neurological disorders or various types of inflammation, as well as processes connected with aging [20,21,22,23]. 

### 2.2. Carotenoids and Oxidative Stress

Exogenous antioxidants, such as α- and β-carotene, lutein and astaxanthin play important roles in preventing oxidative damages of free radical by scavenging activity. For this reason, the most important bioactivity of carotenoids in living organisms is the antioxidant response and oxidative stress resistance [24,25]. They are principal actors of the defense process in marine and terrestrial plants, but their lipophilic nature allows them to penetrate through cellular bilayer membrane and cross the blood-brain barrier, carrying out its biological function also in different regions of human body, including the brain.

Among carotenoids extracted from marine organisms, the red pigment astaxanthin possesses strong scavenging activity against free radicals and other prooxidant molecules, protecting lipid bilayer from peroxidation. This marked effect is due to its unique molecular structure (characterized by polar ionic rings and non-polar conjugated carbon–carbon bonds), which confers to astaxanthin an antioxidant property 10-fold greater than other carotenoids, such as lutein, canthaxanthin, and β-carotene [26]. In particular, polar extremities (ionic rings) confer the potent capacity for quenching free radicals, while the central part of the molecule provides the strong antioxidant potential, due to ability to remove high-energy electrons from free radicals and “delocalize” their electronic energy [26]. Astanxanthin is also well-known to prevent peroxidation at the lipid membrane level with an efficiency greater than all other known antioxidant compounds [27]. Astaxanthin molecule is not subjected to chemical modification during quenching activity, for this reason it can be active for many cycles [28]. From the chemical point of view, this protective effect is due to its ability to scavenge superoxide anion radicals (O_2_^−^) [29]. Moreover, astaxanthin indirectly avoid the activation of the nuclear transcriptional factor NkfB, exerting his protective effect against harmful molecules, such as H_2_O_2_. The neutralization of H_2_O_2_ also prevents the release of cytokines. Speranza and collaborators [30] investigated the in vitro effect of marine astaxanthin on cytokines secretion in human monocytes U-937, stimulated with oxidative agent. H_2_O_2_ is known to induce inactivation of phosphatase-1 (SHP-1), which is a negative regulator of cytokine release. The results showed that 10 μM of astaxanthin was able to reduce proinflammatory cytokines, such as IL-1β, IL-6 and TNF-α, previously induced by treatment with 100 μM of H_2_O_2_. This effect is due, at molecular level, to increased levels of SHP-1 protein and a reduction of NfkB nuclear expression [31]. Superoxide dismutase (SOD) and catalase (CAT) are main intracellular enzymatic targets of astaxanthin. SOD catalyzes the dismutation of O_2_^−^ into stable oxygen molecules (O_2_) and hydrogen peroxide (H_2_O_2_), while CAT promotes the decomposition of H_2_O_2_ to a water molecule and O_2_ [31]. A recent study [32] demonstrated the antioxidant and protective effect of astaxanthin in specific areas of the mice brain (frontal cortex, striatum, parietal cortex, hypothalamus, hippocampus and cerebellum). Researchers focused the attention on specific biochemical non-enzymatic oxidative markers, such as malondialdehyde (MDA), nitric oxide (NO), advanced protein oxidation product (APOP) and glutathione (GSH). They also took into account the biochemical activity of enzymatic antioxidants, such as CAT and SOD. 2 mg/kg/day (body weight) of astaxanthin for 4 weeks improved all types of oxidative markers in both young and aged animals in specific brain regions. The study also clearly demonstrates that the protective effect was greater in young mice than in the old one, resulting in an age-dependent effect. In addition to the brain, testicles are another target organ extremely sensitive to oxidative injuries, which could create a reduction of testosterone production. In fact, some investigations revealed that astanxanthin has a strong antioxidant activity on Leydig cells [33]. These cells were in vitro injured with hydrogen peroxide causing block of testosterone and progesterone production and reduction of steroidogenic acute regulatory protein (StAR). One-hour pre-treatment with 10 μg/mL astaxanthin (before the oxidative injury) was able to restore progesterone production and expression of mature StAR protein in testicular cells [33].

Fucoxanthin belongs to the class of xanthophylls and is a common carotenoid in brown seaweeds. The molecular structure is characterized by an unusual allenic bond, an epoxide group and a conjugated carbonyl group in a polyene backbone, which is responsible of the strong antioxidant activity [34]. Previous studies indicated that fucoxanthin extracted from brown algae *Sargassum siliquastrum* showed a strong in vitro antioxidant and protective effect against oxidative stress [35]. In detail, Vero cells (kidney epithelial cells) were treated with increasing concentrations of fucoxanthin (5, 50, 100 and 200 µM) and then injured with 50 µM of H_2_O_2_ for 24 h. The pre-treatment with the carotenoid was able to prevent the cytotoxic effect of oxidative agent in a dose-dependent manner (63.6%, 69.4%, 78.5%, and 89.2% of cell viability, respectively). Fucoxanthin also exerted a protective effect against H_2_O_2_-mediated DNA damage, in terms of reduction of DNA fragmentation index (63.4%, 52.5%, and 38.8% at the concentrations of 5, 50, and 200 µM, respectively, by comet assay). In addition, fucoxanthin showed a similar protective effect on human fibroblasts against UV-B radiation [36]. Cells treated with only UV-B rays (50 mJ/cm^2^) exhibited a reduction of their viability (43%), while cells pre-treated with 5, 50 and 100 µM of fucoxanthin showed a higher percentage of viability (60%, 77% and 82%, respectively). The same carotenoid pre-treatments were able to significantly reduce the DNA damage: fucoxanthin (5, 50 and 250 µM), indeed, reduced UV-B-induced DNA fragmentation index to 40%, 35% and 20% (obtained by comet assay).

Sangeetha and collaborators analyzed in vivo properties of fucoxanthin compared with β-carotene [37] by feeding rats affected by retinol deficiency with fucoxanthin or β-carotene (0.83 µM and 0.81 µM, respectively). The two carotenoids decreased lipid peroxidation and enhanced CAT and GST activities, showing protective effect against Na^+^K^+^ATPase activity. However, fucoxanthin exhibited greater antioxidant and protection properties than β-carotene.

Mytiloxanthin is a metabolite of fucoxanthin found in tunicates and shellfish. This natural compound has been shown to own a high quenching activity of singlet oxygen (62%) comparable to that of astaxanthin (60%) [38]. This strong effect is probably due to its 11-conjugated-double-bond polyene system and carbonyl and hydroxyl groups that enhance its antioxidant properties [39,40]. Maoka and colleagues [38] also showed a stronger inhibitory activity of mytiloxanthin on lipid peroxidation (20%, formation of LOOH) than fucoxanthin (32%), astaxanthin (24%) and β-carotene (38%), at the same of final concentration (167 μM).

Another marine carotenoid with interesting antioxidant effect is zeaxanthin [26]. This natural compound efficiently scavenges ROS and protects proteins, lipids, or DNA from oxidative damage by regulating other antioxidant processes and augmenting the production of endogenous antioxidant compounds such as intracellular GSH. The antioxidant properties of zeaxanthin are similar to those of α-tocopherol. Indeed, the assimilation of zeaxanthin or α-tocopherol reduces oxidized glutathione (GSSG), increases the levels of intracellular reduced glutathione together with the GSH:GSSG ratio, in response to oxidative stress [41].

In the last decade, two rare marine monocyclic carotenoids such as saproxanthin and myxol have been isolated by bacteria belonging to the family *Flavobacteriaceae*. These carotenoids show strong antioxidant activity, lipid peroxidation prevention in brain, and neuroprotectiion against L-glutamate toxicity [26,42]. The antioxidant activity of saproxanthin and myxol is even stronger than that of zeaxanthin and β-carotene as revealed by Shindo and colleagues, who tested the lipid peroxidation inhibitory activity of saproxanthin, myxol and zeaxanthin in the rat brain homogenate [43]. Saproxanthin showed the strongest effect with an IC_50_ of 2.1 μM, compared to myxol and zeaxanthin (6.2 and 13.5 μM, respectively). The two rare carotenoids showed lower IC_50_ (3.1 and 8.1 μM, respectively) than astaxanthin and β-carotene (>500 and >100 μM, respectively), when their protective effect was tested against L-glutammate toxicity on N18-RE-105 cell line (embryonic rat retinal neuron hybrid cells) as they produce a stabilization of biological structures, inducing a reduction of membrane permeability to oxygen.

There is also in vivo evidence for the biological roles of carotenoids against oxidative stress. For instance, Allard et al. [44] supplemented smokers with 20 mg of β-carotene and showed, after 4 weeks, a decrease in pentane levels in their breath (an indicator for monitoring lipid peroxidation) down to the levels of non-smokers individuals.

Although the beneficial properties of carotenoids have been always proposed as the main mechanism of action, recent studies are describing these natural compounds as prooxidant agents, able to increase the total radical yield in a biological system [45,46]. There are some chemical and physical conditions, such as high concentration of bioactive compounds or oxygen partial pressure, which could determine the switch of carotenoids, from antioxidants to prooxidants [47]. In fact, high levels of the oxygen partial pressure can induce the reaction between a carotenoid radical and molecular oxygen, generating a carotenoid-peroxyl radical, which is able to act as a prooxidant agent, by promoting oxidation processes of unsaturated lipids.

### 2.3. Carotenoids and Age-Related Diseases

Aging is a physiological process that starts at conception and continues as long as we live. Such a relentless phenomenon is influenced by genetic components and environmental factors. Cardiovascular apparatus (discussed in the Section 2.5), skin and nervous system (brain and spinal cord) are compartments of the human body mainly affected by the aging process and therefore by age-related diseases.

Skin is our largest organ, which being mainly exposed to chemical and physical factors contributing to the insurgence of age-related diseases, represents a fundamental barrier between our body and the external environment. One of the most dangerous environmental factor is UV radiation deriving from both natural and artificial sources, which can directly affect human skin, causing epidermal and dermal damages, photosensitivity disorders, and/or the formation of free radicals (mainly ROS) [48]. Dermal tissue has developed complex defense mechanisms against the harmful effects of these radiations and their related reactive products. This protective system needs, for a proper functioning, availability of exogenous antioxidant substances such as carotenoids in dermal tissue. The specific carotenoids commonly present in human skin include lycopene, lutein, α-, γ-, β-carotene, zeaxanthin and their isomers [49]. The antioxidant defense system in human skin involves several compounds, working with synergistic effect. For instance, previous studies reported that the antioxidant effects in cultured human fibroblasts, irradiated with UV-A light (20 J/cm^2^), were enhanced when a combination of antioxidant compounds (with β-carotene, as the main component, α-tocopherol and ascorbic acid) was used as a treatment [50]. In addition, a mixture of β-carotene and vitamins E and C showed a stronger synergistic scavenging effect of the reactive nitrogen species than the single antioxidants [51].Recent investigations demonstrated that the green microalga *Tetraselmis suecica* is characterized by a natural pool of bioactive pigments (including neoxanthin, violaxanthin, antheraxanthin, lutein, α- and β-carotene) working synergistically against oxidative damage [52]. In particular, this pool of bioactive pigments showed scavenging activity (with DPPH assay) and repairing activity when the *T. suecica* crude extract was used as recovery treatment in human fibroblasts after pre-injury with H_2_O_2_ (30 mM for 1 h). Moreover, *T. suecica* crude extract was also able to significantly reduce the Prostaglandin E2 release, induced by same oxidative injury. Carotenoids have been also demonstrated to be efficient for the cure of erythema formation [53], since β- carotene supplementation (30 to 90 mg/day) is responsible of protection against sunburn (in a dose-dependent manner, after 24 weeks of treatment) [54]. Moreover, carotenoids are able to reduce the aging process at the skin level. In fact, investigations based on optical skin surface topography revealed that individuals with high dermal concentration of carotenoids showed a “skin age” lower than in individuals with low carotenoid concentrations, and looked younger [55].

One of the most severe age-related disease is Age-related Macular Degeneration (AMD), which is the leading cause of blindness in the developed countries. GSH is a reducing compound present in the lens at high concentrations and due to its antioxidant properties is essential to maintain tissue transparency [41]. This compound decreases in old lenses, giving rise to oxidative and degenerative processes. Zeaxanthin and lutein cover key roles in the protection from AMD, since their intake is associated with the increase of the macular pigment density, acting on intracellular levels of GSH [26]. In particular, these carotenoids possess a plethora of antioxidant properties, such as ROS scavenging, protection from DNA damages and from protein and lipid oxidation. An in vitro study performed on Human Dermal Lymphatic Endothelial Cells (HLEC) demonstrated the ability of lutein and a zeaxanthin to reduce oxidative damage [56]. Indeed, a pre-incubation step of cells with 5 µM of lutein or zeaxanthin for 48 h drastically reduced the oxidative damage in DNA, lipids and proteins due to 100 µM of H_2_O_2_ with an overall improvement of intracellular redox status.

Many epidemiological studies have already demonstrated the direct association between intakes of carotenoids and AMD insurgence [57,58]. In particular, in a prospective study, Wu et al. [59] demonstrated that a high intake of bioavailable lutein/zeaxanthin is associated with long-term reduced risk of advanced AMD in individuals aged 50 years or older.

### 2.4. Carotenoids and Cancer

In the last two decades, researchers have described a plethora of beneficial effects generated by a diet rich in fruits, vegetables and other food containing carotenoids for the human body; a sufficient daily intake of healthy food contributes to significantly reduce the risk of cancer insurgence and development. Several carotenoids, such as astaxanthin, fucoxanthin, lutein, α-carotene and lycopene may reduce the risk and decelerate progression of cancer [60,61]. For this reason, in the last decade, there has been increasing scientific interest in carotenoids and their natural sources, due to their antioxidant potential that produces enhancement of the immune system machinery, a key factor in the elimination of carcinogenic injury [62]. There are many studies showing an antiproliferative effect of marine carotenoids on different cancer cell lines, highlighting molecular targets and their effects on human population.

Fucoxanthin represents one of the most abundant carotenoids in nature, especially in marine environment; it contributes to about 10% of the total natural carotenoid production [63].The seaweed *Undaria pinnatifida* is on the food market of several countries, such as Japan and Korea [64]. It was one of the first marine sources of fucoxanthin to be investigated for its antiproliferative effect. In particular, fucoxanthin was in vitro tested on several human cell lines, such as leukemia cells (HD-60) and epithelial colorectal adenocarcinoma cells (Caco-2, DLD-1 and HT-29). Cell viability strongly decreased in a dose-dependent manner when HL-60 cells were treated with 11.3 and 45.2 μM of fucoxanthin (46% and 17% of viable cells, respectively) [65]. Ca. 15 μM of fucoxanthin for 72 h is able to induce a significant decrease of cell viability in Caco-2, DLD-1 and HT-29 (15%, 29% and 51%, respectively). The antiproliferative effect was related to the activation of apoptotic death, creating a typical morphological apoptotic features and characteristic DNA fragmentation [66]. Moreover, fucoxanthin was tested also on other normal and cancer cell types, such as normal human embryonic lung fibroblasts (MRC-5), human umbilical cord fibroblasts (HUC-Fm), mouse melanoma (B16), human colorectal carcinoma (HCT116) and human prostate cancer (PC-3) [67,68,69]. This panel of cell lines was treated for 72 h with two different concentrations of fucoxanthin (5 and 10μM) and surprisingly the marine carotenoids did not affect the normal cell viability (MRC-5). Moreover, fucoxanthin exhibited an antiproliferative effect, inducing a strong decrease of HCT116 and PC-3 cell viability at 5 μM (35% and 65%, respectively) and 10 μM (30% and 40%, respectively).This marine carotenoid has many apoptotic intracellular targets, such as antiapoptotic factor Bcl-xL, poly-ADP-ribose polymerase (PARP), caspase 3 and 7. In detail, fucoxanthin promotes cleavage of caspases and decreases the expression level of Bcl-xL, inducing the apoptotic cascade [70]. Moreover, fucoxanthin acts on cyclin D and JAK/STAT (Janus Kinase/Signal Transducer and Activator of Transcription) signal pathway, inducing the decrease of gene expression. Other target genes are SAPK/JNK, p38 MAPK and GADD45a [71,72,73,74].

Fucoxanthinol, a fucoxanthin metabolite, was also studied for its potential inhibitory activity on PC-3 proliferation. While fucoxanthin exhibited an IC_50_ (50% inhibitory concentration) of 3.0 μM, the IC_50_ of fucoxanthinol was 2.0 μM [67].In addition to PC-3, 20 μM of fucoxanthin reduced cell viable percentage to 5% of other prostate cancer cells, such as human prostate carcinoma cells (DU 145) and androgen-sensitive human prostate adenocarcinoma cells (LNCaP) [75]. A similar antiproliferative effect has been observed for fucoxanthin extracted from brown seaweed *Laminaria japonica* on bladder cancer. In particular, 20 μM of fucoxanthin drastically reduced the proliferation rate of human urinary bladder cancer cells (EJ-1), producing 93% of apoptotic cells after 72 h with respect to untreated cells [76].

Another fucoxanthin metabolite that was investigated for its potential anticancer activity is halocynthiaxanthin, which can be isolated from sea squirt or sea pineapple. This pigment exerted greater cytotoxic effect on human neuroblastoma cells (GOTO) than fucoxanthin [77]. In particular, 5 μg/mL ofhalocynthiaxanthininduced cell death in 100% of GOTO, whereas the same concentration of fucoxanthin produced a reduction of 12% of viable GOTO cells. These studies clearly demonstrate the relevant potential of fucoxanthinmetabolites. The sea pineapple *Halocynthiaroretzi* is a source of fucoxanthinol and halocynthiaxanthin. Konishi and collaborators [78] examined the effect of these two fucoxanthinmetabolites on human breast adenocarcinoma cell line (MCF-7), HL-60 and Caco-2. HL-60 cell line was the most affected in terms of viability percentage, since after 72 h of incubation halocynthiaxanthin completely inhibited cell growth (0% of viable cells) and fucoxanthinol drastically decreased the cell viability to 2%. Halocynthiaxanthin induce a decrease to 70% of cell viability in MCF-7, while fucoxanthinol of about 15% on same cell line. Caco-2 cells responded similarly to halocynthiaxanthin and fucoxanthinol, decreasing cell viability (15% of viable cells after both treatments respect to untreated control).

In a recent study, Rokkaku et al. [79] demonstrated the effects of carotenoids on the cell viability of different osteosarcoma cell lines. Fucoxanthin and fucoxanthinol were able to reduce cell viability of all four osteosarcoma cell lines depending on the dose considered. On the other hand, the antiproliferative effect of other carotenoids, β-carotene and astaxanthin, was less significant, although β-carotene reduced LM8 (murine osteosarcoma) cell viability at highest concentration.

Another natural carotenoid with antiproliferative activity is canthaxanthin, which can be extracted from crustaceans and algae. Many studies provide evidence of anticancer potential of canthaxanthin on different type of human tumors. In particular, this carotenoid has been tested on human colon adenocarcinoma cells (WiDr), human and murine melanoma (SK-MEL-2, JB/MS and B16F10) and fibrosarcoma cells (PYB6) [80,81]. WiDr treated with 10 μM of canthaxanthin for 48 h decreased cell viability to 18%, while the same treatment on SK-MEL-2 induced a reduction to 20% of viable cells. Moreover, 100 μM of canthaxanthin drastically reduced the growth of JB/MS, B16F10and PYB6. Canthaxanthin did not show cytotoxic effect on normal cells when non transformed mouse embryonic fibroblast cells (NIH-3T3) were treated with the specific carotenoid. This inhibitory effect of canthaxanthin on tumor cells was mediated by TNF-α [82]. A study conducted by Shklarand Schwartz [83] clearly demonstrated in vivo activation of TNF-α in macrophages collected from tissues surrounding hamster buccal pouch carcinoma, following the local injection of 1.9 mg/mL of chantaxanthin with other bioactive pigments; this effect could be related to immune system stimulation. Another study described in vitro activity of T- and B-lymphocyte responses, isolated from rat model treated with 2 g/kg of canthaxanthin, which resulted enhanced in animals fed with carotenoid diet compared to control group [84].

Dinoflagellates represent a potential source of carotenoids with strong antiproliferative activity. For instance, the dinoflagellate *Heterocapsa triquetra* produce peridinin [85], which is able to induce apoptosis in colorectal adenocarcinoma cell line (DLD-1). In detail, 20 μM of peridinin induced a decrease of viable cells to 40% compared to untreated cells after 72 h. The marine carotenoid induced chromatin fragmentation and up-regulation of caspase-9 and caspase-8 when DLD-1 are treated for 48 h with 20 μM of peridinin.

Astaxanthin has been investigated for its anticancer property. In particular, Astaxanthin (10 μM) interfered with androgen-induced proliferation of human prostatic adenocarcinoma (LNCaP) in a dose-dependent manner and reduced the prostate specific antigen (PSA) secretion of 25% and intracellular PSA level of 50% [86].Moreover, Astaxanthin can act as anticancer compound enhancing the immune system response. In a study carried out by Jyonouchi and collaborators [87], astaxanthin exhibited anticancer effect against transplantable methylcholanthrene-induced fibrosarcoma (Meth-A tumor) cells. Astaxanthin (0.02%, 40 mg/kg/day) was used in the diet of BALB/c mice for three weeks before the induction of tumorigenesis. The researchers clearly showed the lower tumor size and weight in mice fed with astaxanthin respect to the control group. The same treatment induced enhancement of cytotoxic T lymphocyte activity and interferon-g (IFN-γ) production.

Siphonaxanthin, found in edible green algae, exhibited an antiproliferative effect on HL-60 [88]. In particular, 20 μM of siphonaxanthin was able to completely decrease cell viability after only 6 h. This strong inhibitory effect has also been investigated at molecular level. Bcl-2, caspase 3, GADD45α and DR5 were target genes of siphonaxanthin, showing that antiproliferative effect was mediated by an activation of specific apoptotic mechanism.

Results from experimental and epidemiological studies about effects of carotenoids report a reduction of the risk of prostate cancer insurgence [89]. Among various carotenoids, lycopene was demonstrated to be the most powerful effect in the reduction of risk for this kind of tumor, through a significant enhancement of the physiological mechanisms involved in the oxidative stress defenses [90]. Recent case-control studies have described the direct relationship between diet rich in carotenoids and insurgence of lung cancer among non-smokers. These studies confirmed a reduction of lung cancer risk in individuals with a daily intake of food source of α-carotene, lutein, lycopene, β-cryptoxanthin and β-carotene [22]. However, it seems that the correct interaction between biologically active carotenoids and human body is lifestyle-dependent. In fact, a large number of epidemiological studies have investigated and described the negative effects of carotenoids in smokers [91,92]. In particular, smokers have unexpectedly showed an increase of lung tumor rates after high, long-term, β-carotene supplementation. There is a large number of hypotheses trying to explain the chemical and molecular mechanism of this undesired effect of carotenoids in tobacco smokers. Some hypotheses suggest alteration of signaling pathways and retinoid metabolism, dysfunction of interaction with Cytochromes P450 (CYPs) enzymes and deregulation of prooxidation/DNA oxidation due to β-carotene intake [93]. In summary, several studies on humans and animals have demonstrated that specific environmental factors and heavy smoking seem to influence or even invert the effect of carotenoids. In contrast, many evidences of beneficial effects of carotenoids are reported in the normal population, with a healthy lifestyle.

### 2.5. Carotenoids and Atherosclerosis-Related Cardiovascular Diseases

One of the most studied free radical-mediated disorders is atherosclerosis, (or arteriosclerotic vascular disease), which remains a major cause of morbidity and mortality in developed countries. Atherosclerosis is a pathological condition where the arteries reduce their diameter and lose their flexibility due to an excessive build-up of plaques around the artery walls, leading to heart attack and stroke. Atherosclerosis starts when the endothelium is damaged in some point and Low-Density Lipoprotein Cholesterol (LDL-C) accumulates in the artery wall. Moreover, it is already well recognized that atherosclerosis is directly related to inflammatory process, suggesting, as adjuvant therapeutic strategy, use of natural compounds for the inflammation modulation. In addition, free radical compounds and consequent oxidative stress facilitate pathogenesis of atherosclerosis, since oxidative modification of LDL-Cis crucial for the initiation and progression of the pathology [94] that includes the inflammatory process, endothelial dysfunction and vascular remodeling [95]. An increased concentration of ROS has been also associated with a functional inactivation of nitric oxide (NO), an effect generated by reaction of these reactive species with superoxide anion (O_2_^−^). Subsequent to the interaction ROS/O_2_^−^, there is a large peroxynitrite (ONOO^−^) production that causes reduction in vascular NO bioavailability (ROS oxidize cofactors of the NO synthase, reducing their active forms and consequently leading to a decreased NO production), a characteristic condition of the early stage of atherosclerosis [96].

Many studies have been carried out in the last years on the possible correlation between antioxidant compounds intake, reduction of oxidative stress and decreased risk of cardiovascular diseases [96,97,98]. To date, results coming from basic research and clinical studies have not provided consistent answers. Some studies, indeed, describe the beneficial effects of some carotenoids reducing cardiovascular diseases, other investigations did not observe any correlation or even reported an inverse relationship between antioxidant compounds and reduction of cardiovascular diseases. However, there are a huge amount of epidemiological studies that suggest a lower insurgence of cardiovascular diseases in the Mediterranean countries compared to the rest of Europe and the United States [97]. This reduction of cardiovascular-related mortality could be in part due to the typical Mediterranean diet, largely characterized by food rich in carotenoids, such as vegetables and fruit. In addition to epidemiological data, there are also in vitro and in vivo studies supporting the thesis of the beneficial role of carotenoids in the prevention of cardiovascular diseases. It has been recently demonstrated that β-carotene and lycopene (0.02 and 0.5 µmol/L, respectively), added before and after the proinflammatory injury, reduced the inflammatory response in TNF-α-treated human umbilical vein endothelial cells (HUVECs) [98]. The TNF-α treatment, indeed, induced monocyte (U937)-endothelial adhesion, which is inhibited by β-carotene and lycopene. Moreover, the two carotenoids reduced the expression levels of genes coding for vascular adhesion proteins, such as VCAM-1, ICAM-1 and E-Selectin. The observed effect could be explained through the redox balance regulation of these carotenoids, able to maintain the NO bioavailability [96,98]. Other in vitro [99] and in vivo [100,101] studies support these results. In particular, Monroy-Ruiz and collaborators [100] demonstrated that astaxanthin improved endothelial function on resistance arteries. In addition to this effect, they observed a decrease in oxidative stress and an improvement in NO bioavailability.

Obesity, a disorder of energy balance causing activation of inflammation, represents another important factor involved in the pathogenesis of atherosclerotic process, since there is link between chronic inflammation and cardiovascular diseases. For this reason, many studies have been carried out to discover new natural compounds and their anti-obesity mechanisms of action that interfere with energy balance. A recent study investigated the effect of fucoxanthin (extracted from *U. pinnatifida*) and its hydrolyzed product fucoxanthinol on the absorption of triglycerides in rats [102]. The two marine carotenoids (2 mg) were able to drastically reduce the lymph triglyceride absorption by 50% after 4 h. This effect was tightly related to the pancreatic lipase inhibitory activity of fucoxanthin and fucoxanthinol in the intestinal lumen.

The first randomized study describes the effect of astaxanthin intake in humans [103]. Subjects (61 non-obese individuals) were fed with 0, 6, 12 and 18 mg/day of astaxanthin. After 12 weeks, LDL-C significantly increased with lower doses of astaxanthin (6 and 12 mg) and the individuals showed serum triglyceride reduction, mainly with higher concentration of the carotenoid (12 and 18 mg). The study also demonstrated the increase of serum adiponectin levels with doses of 12 and 18 mg of astaxanthin, suggesting that this natural compound was able to influence triglyceride and HDL-C in relation to augmented adiponectin in humans. One of the first reports of the effect of astaxanthin on the cardiovascular system was carried out by Hussein and collaborators [104]. Short-term (2 weeks) oral administration of astaxanthin (50 mg/kg) induced a strong decrease of arterial blood pressure (BP) in spontaneously hypertensive rats (SHR). The same carotenoid treatment, but for 5 weeks, had the same effect also in stroke prone SHR (SHR-SP). The in vitro study demonstrated that the NO is the main target of astaxanthin (which induces reduction of NO gene expression) and the possible key factor for the reduction of BP. Astaxanthin is also able to influence LDL oxidation [105]. In particular, astaxanthin (12.5, 25 and 50 μg/mL) can strongly prolong the oxidation lag time (31.5, 45.4, 65.0 min), in a dose dependent manner, when LDL are in a reaction system together with the bioactive pigment and lipid-soluble azo initiator (2,2′-Azobis[4-Methoxy-2, 4-Dimethylvaleronitrile], typically used for in vitro studies on lipid peroxidation). This study clearly demonstrated that the carotenoid intake can significantly inhibit LDL oxidation, contributing to the prevention of atherosclerosis.

Lutein has been described as a protective compound against the early stages of the atherosclerotic process. In vitro, in vivo and epidemiologic investigations have been carried out by Dwyer and collaborators [106]. They have demonstrated that lutein (0.1, 1, 10 and 100 nM) produced a strong dose-dependent reduction in chemotactic signal for monocytes in the co-culture model of lipoprotein oxidation with the artery wall. In this way, lutein was able to inhibit the inflammatory response of monocytes to LDL associated with artery wall. Moreover, this effect was confirmed in apoE-null mice and LDL receptor–null mice, where an enriched lutein diet produced a significant reduction of atherosclerotic lesion size in the aortic arch. The study also demonstrated the correlation between high plasma lutein levels and progression of intima-media thickness in 480 individuals. However, as mentioned before, other in vivo studies showed that carotenoid intake does not positively influence the risk of ischemic heart disease and stroke. In particular, the supplementation of 20 mg/day of β-carotene long term (over six years) produced no significant variation of cardiovascular-related mortality [107]. Further investigations have demonstrated an increase of post-trial risk of a first nonfatal myocardial infarction [108], although other studies had already reported that a supplementation of a larger amount of β-carotene (50 mg/day) to non-smoking males and female adults did not produce a significant increase of cardiovascular-related mortality [109]. Moreover, the Heart Protection Study did not provide confirmation of a direct relation between β-carotene intake and the five-year mortality after supplementation or incidence variation of any kind of cardiovascular diseases [110]. More data and evidence are needed to clarify the reason for the divergence among these studies and to better elucidate the biological effect induced by carotenoids on structures and processes involved in atherosclerosis-related cardiovascular diseases.

The biological effects and the main targets of natural carotenoids described in the Section 2 are summarized in Table 1.

### 2.6. Carotenoids and Bioavailability

Although many interesting biological activities, bioavailability of carotenoids is lower than that of other fatty components, such as α-tocopherol and triacylglycerol. During the digestive process, carotenoids should be solubilized via several steps before uptake by intestinal epithelial cells can occur [111]. In this step, dietary lipids facilitate carotenoid dispersion. Carotenoids are dissolved into dietary lipids and then dispersed as an emulsion in the digestive fluid. Digestion of the dietary lipids in the emulsion occurs thanks to lipolytic enzymes and bile fluid, and finally carotenoids are solubilized in mixed micelles. Carotenoids incorporated into mixed micelles are thought to become accessible to uptake by the intestinal epithelial cells. For this reason, the bioaccessibility is defined as the ratio of carotenoids solubilized in the mixed micelles to the total carotenoids ingested. The bioaccessibility is an important factor for bioavailability [112]. Many marine-derived products in the nutraceutical industry are composed of oils and a wide range of marine organisms, such as fungi, microalgae, macroalgae and krill represent rich sources of polyunsaturated fatty acids [113].

## 3. Sources of Carotenoids from Marine Organisms

Carotenoids are widespread in nature and, in general, all colored terrestrial vegetables and fruits are sources of these compounds [114]. However, also a wide range of marine organisms including algae, animals and microorganisms such as microalgae, cyanobacteria, fungi, heterotrophic bacteria and archaea are known to produce carotenoids [9]. Among them, microalgae are considered the main sources of carotenoids for industrial use, but other organisms could be valid alternatives for their employment at large scale in cosmetic and pharmaceutical fields [2,6].

### 3.1. Marine Algae and Seagrass

Microalgae live in the sunlight zone of all existing marine ecosystems, from the ice of the polar regions to the coral reefs of the tropical areas, and in other aquatic systems. These algae provide the food supporting marine trophic webs. They have evolved a wide range of photoprotective pigments, which cover most of the visible light wavelengths available in underwater habitats [115], and are characterized by a chemo-diversity reflecting the different photic conditions of marine ecosystems. The number of marine microalgae species estimated so far ranges between 30,000 and 50,000, but the number of undescribed species can range from thousands to millions of species spread across the globe [116]. These organisms have provided a great contribution to the development of products for biotechnological, pharmaceutical and cosmeceutical applications due to their fast growth rate and ability to produce a large variety of bioactive substances. However, only a few thousand strains are kept in collections, and a few hundred have been investigated for chemical content and considered relevant for the market [117,118]. Marine microalgae represent a major natural source of carotenoids (up to 8–14% of their dry weight). They can accumulate carotenoids either as essential pigments for their survival, acting as structural and functional components of the cellular photosynthetic apparatus (primary carotenoids) or after exposure to specific environmental stresses (secondary carotenoids) [119]. Carotenoids synthesized by microalgae include carotenes (α- and β-carotenes, and lycopene) and xanthophylls (e.g., zeaxanthin, lutein, ketocarotenoids, canthaxanthin, echinenone, astaxanthin, violaxanthin, diadinoxanthin, dinoxanthin and fucoxanthin) [120]. The Chlorophyceae family, which includes *Chlorella*, *Chlamydomonas*, *Dunaliella*, *Muriellopsis* and *Haematococcus* spp., is characterized by microalgae with the widest diversity of pigments [121]. These microalgae overproduce secondary carotenoids in response to adverse growth conditions [122]. Among the microalgae, which are already exploited commercially, the different species belonging to the genus *Dunaliella* have received special attention in the production of these high-value compounds. *Dunaliella* has a wide geographical distribution and is able to survive also in extreme environmental conditions [9]. In particular, *Dunaliella salina*, has long been recognized as an efficient biological source of the orange pigment β-carotene [123], which is accumulated in oil globules in the interthylakoid spaces of the chloroplasts depending on environmental conditions (i.e., salinity, temperature, light intensity, and nitrogen concentrations) [123]. When *Dunaliella salina* is exposed to specific extreme environmental conditions, such as very high light intensity and salinity, extreme temperatures and/or nutrient limitation can accumulate up to 10–13% of dry algal biomass as β-carotene [124] other than α-carotene, lutein and zeaxanthin. Thanks to its physiological characteristics and easy techniques of cultivation, *Dunaliella* is used for large-scale production of β-carotene [124].

*Dunaliella salina* is also considered an excellent alternative to conventional plant sources for obtaining lutein, as well as microalgae belonging to the genus *Chlorella* (e.g., *C. protothecoides*, *C. pyrenoidosa, C.* *zofingiensis*) [125,126] and *Scenedesmus* [127,128], generally used in large-scale cultures for commercial production [129].

*Tetraselmis suecica* is a marine green microalga belonging to the class Chlorophyceae, widely used in aquaculture for feeding mollusks and crustacean larvae and as a probiotic in fish farms. *T. suecica* is rich in tocopherol, carotenoids and chlorophyll and has been suggested as a food supplement in human and animal diets. The total carotenoids content of *T. suecica* exhibited antioxidant and repairing activity in human cell lines [52].

The microalgae *Hematococcus pluvialis* has been widely studied for more than two decades for the production of the red pigment astaxanthin [130] (and references therein), the main isomer of which is identical to that present in wild salmon [131]. Additionally, some species of microalgae can be efficiently genetically engineered to obtain transgenic microalgae, allowing us to enhance the productivity of natural compounds [132].

Macroalgae have a long tradition as a food source in Asian countries, and some of them are cultivated in different countries, including Japan, Korea and China, thus potentially representing renewable and sustainable resources for pharmaceutical, cosmeceutical and nutraceutical applications. Seaweeds (macroalgae) represent an important source of carotenoids, such as fucoxanthin, β-carotene, lutein, and siphonaxanthin. They are generally localized in the chloroplasts, accumulated in vesicles, cytoplasmic matrix or bound to membranes and other macromolecules in the intracellular space [133]. In particular, fucoxanthin represents a component of the light-harvesting complex for photosynthesis and photoprotection [133], and is present in several brown macroalgae, which are almost entirely marine and widely distributed in cold and temperate ecosystems throughout the world. Some species of brown seaweeds, such as the giant kelp *Macrocystis pyrifera*, forms extensive seaweed forests in coastal areas of the north Pacific Oceans, creating an important habitat for several other organisms. Brown algae producing fucoxanthin include *Hijikia fusiformis* [134], *Undaria pinnatifida* [135], *Sargassum* sp*.* [136], *Laminaria* sp*.* [137] and *Fucus* sp*.* [138]. Fucoxanthin has been also isolated for bioactivity studies from other marine seaweeds such as: *Alaria crassifolia*, *Cladosiphon okamuranus*, *Cystoseira hakodatensis*, *Eisenia bicyclis*, *Hijikia fusiformis*, *Ishige okamurae*, *Kjellmaniella crassifolia*, *Myagropsis myagroides*, *Padina tetrastromatica*, *Petalonia binghamiae*, *and the diatoms Chaetoseros* sp*.*, *Cylindrotheca closterium*, *Odontella aurita*, *and Phaeodactylum tricornutum* [139] (and references contained therein).

Seagrasses are plants widely distributed along temperate and tropical coastlines of the world and have key ecological roles in coastal ecosystem functioning and in supporting biodiversity [140]. These are exposed to very variable light radiation conditions due to the different depth ranges they colonized [141], and have a photosynthetic carotenoid pool similar to that of terrestrial plants [142]. However, detailed investigations on this pool are limited [143]. Previous studies revealed that four Mediterranean seagrass species, *Posidonia oceanica*, *Cymodocea nodosa*, *Zostera noltii* and *Halophila stipulacea*, contain β,β-carotene, lutein, zeaxanthin, violaxanthin, neoxanthin and siphonaxanthintype pigments [142]. Other investigations reported that the leafs of *Cymodocea nodosa* and *Zostera marina*, contain seven photosynthetic carotenoids, including β-carotene, lutein, lutein epoxide, violaxanthin, antheraxanthin, neoxanthin and zeaxanthin, with diverse physiological functions, from light harvesting to photooxidation prevention [143]. Among plants, mangroves develop along the marine coastlines and transitional ecosystems of tropical and subtropical regions. Information about carotenoids in these plants is very limited. Previous studies investigated the levels of astaxanthin in the leafs of six mangrove species sharing the same brackish water, and revealed significant variation in the concentrations of this carotenoid depending on the species [144]. Biotechnological investigations applied to seagrass and mangrove forests for the exploitation of carotenoids are still very scant. This is probably due to the fact that the scientific/industrial applications of these fundamental components for marine ecosystems, already subjected to human-induced regression, would be not environmentally sustainable.

### 3.2. Prokaryotes (Bacteria and Archaea), Cyanobacteria, Fungi and Fungi-Like Protists

Despite heterotrophic microorganisms not having received the same attention given to the algae as source of carotenoids, also heterotrophic bacteria, fungi (especially pigmented yeasts) and fungi-like protists represent a potentially important source of these molecules, which are synthesized de novo [6]. Among marine heterotrophic microorganisms, bacteria such as those belonging to the genera *Agrobacterium* and *Paracoccus*, generally isolated from coastal ecosystems, are promising producers of astaxanthin [11,145]. Two rare carotenoids with relevant antioxidant action (i.e., saproxanthin and myxol) have been isolated from new strains of marine bacteria belonging to the family *Flavobacteriaceae* [43]. The antioxidant activities of saproxanthin and myxol were even greater than those of the commonly used zeaxanthin and β-carotene [43]. However, these new and rare marine carotenoids require a thorough evaluation before their implementation within cosmeceutical products [146]. Previous investigations revealed that halophilic microorganisms, and in particular, haloarchaea belonging to *Haloferacaceae* family inhabiting high-salinity environments, such as salt marshes or hyperhaline ponds can be considered good producers of carotenoids. In particular, these halophilic organisms produce phytoene, β-carotene, lycopene, derivatives of bacterioruberin, and salinixanthin [147]. Recent scientific evidence has indicated an improved production of carotenoids by *Haloferax* *mediterranei*, which can accumulate high concentrations of bacterioruberin, known to exhibit a better free radicals’ scavenging ability than β-carotene [148].

Cyanobacteria are photosynthetic microorganisms commonly found in diverse aquatic environments, including marine ecosystems. The most abundant carotenoids produced by marine cyanobacteria are carotenes (e.g., β-carotene) and various types of xanthophylls such as synechoxanthin, canthaxanthin, caloxanthin, echinenone, myxoxanthophyll, nostoxanthin, zeaxanthin [149]. Marine cyanobacteria that are technologically interesting and with high potential for the production of carotenoids (e.g., of canthaxanthin) are those belonging to genera Anabaena and *Nostoc* [150].

Several yeast species isolated from marine environment synthesize carotenoids. In particular, the genera *Xanthophyllomyces*, *Rhodotorula* and *Phaffia* have been used to obtain astaxanthin [11]. Although yeasts and bacteria produce lower amounts of astaxanthin than algae, they have faster growth rates and easier cultivation techniques [7,151].

Even fungi-like protists such as thraustochytrids are producers of carotenoids. They have a wide geographical distribution from the polar to tropical regions, and include planktonic and benthonic forms inhabiting various habitats spanning sediments of mangroves, estuaries and deep-sea ecosystems [152]. In particular, *Thraustochytrium* strains ONC-T18 and CHN-1, Thraustochytriidae sp. AS4-A1 (*Ulkenia* sp*.*) and *Aurantiochytrium* sp. KH105 synthesize different carotenoids including β-carotene, astaxanthin, zeaxanthin, cantaxanthin, phoenicoxanthin and echinenone [10]. Engineering approaches have allowed an increase in production of carotenoids (even nine-fold increased astaxanthin content production) such as in the case of *Aurantiochytrium* sp. SK4 [153]. Therefore, the development of genetic tools and genomic sequencing applied to thraustochytrids is fundamental to expand our knowledge of the sources of carotenoids to be used in biotechnological field.

### 3.3. Marine Animals

Marine animals (generally invertebrates) contain a wide range of carotenoids that show a great structural diversity [2]. Despite the fact that they do not synthesize carotenoids de novo, these pigments can be accumulated directly by food intake or obtained through metabolic transformations [154]. Carotenoids play vital roles in marine animals including the photoprotective and antioxidant functions via light energy dissipation, and free radical detoxification, especially in shallow habitats, where periodic exposure to excessive solar radiation and harmful UV rays occurs [155,156]. Carotenoids are also important in the nutrition of animals, having roles in provitamin A activity and immunological modulation [157].

Among marine invertebrates, sponges, anemones, corals, jellyfish and ascidians, as revealed from their bright colors, are the marine invertebrates exhibiting the widest range of pigments.

Sponges contain more than forty carotenoids [158], and have adaptations to a wide spectrum of marine habitats from the Antarctic sea-ice to tropical reef ecosystems. Typically, sponges contain aryl carotenoids such as isorenieratene, renieratene and renierapurpurin [159]. Sponges accumulate pigment granules in the amebocytes (i.e., specialized cells involved in the defense of the organism against pathogens, feeding and/or other roles) or are produced by their symbionts (microalgae or bacteria). Novel carotenoids assumed to be metabolites of fucoxanthin originating from microalgae have been isolated from *Ianthella basta* and *Prianos osiros* [159]*.*

Cnidaria accumulate several pigments, including carotenoids [160]. Coral reefs are among the most diverse biological communities on the Earth and represent relevant sources of natural molecules for the human being [161]. Most corals obtain the majority of their energy and nutrients from photosynthetic dinoflagellates of the genus *Symbiodinium* that live within the coral’s tissue [162]. Such symbiotic microalgae mainly contain pigments such as peridinin, pyrrhoxanthin, and diadinoxanthin [161]. However, also zeaxanthin, lutein, and, fucoxanthin can be accumulated by corals due to their associations with cyanobacteria and epizoic and/or endolithic algae [163]. The major role of carotenoids in corals is the protection of the symbiotic algae from irreversible light-induced photoinhibition that may lead to their loss or expulsion from corals [164]. Peridinin and pyrroxanthin can play important roles in reproduction in corals, as for the astaxanthin in salmonid fishes [165]. Peridinin and pyrrhoxanthin were found to be more relevant carotenoids also in tridacnid clams [166]. Previous studies reported that the sea anemones *Actinia equina* and *Tealia feline* accumulate unique carotenoids such as 2-nor-astaxnthin and actinioerythrin, while *Anemonia sulcata* contains peridinin [156].

Molluscs contain a wide diversity of carotenoids, which reflect their different trophic strategies. The principal carotenoids in shellfishes include β-carotene, lutein A, zeaxanthin, diatoxanthin, alloxanthin, astaxanthin, pectenolone, pectenol A, mytiloxanthin and mactraxanthin [156]. Bivalves are filter feeders and obtain carotenoids by feeding on microalgae or modifying them through metabolic reactions. They generally contain metabolites of fucoxanthin, peridinin, and diatoxanthin [160]. Chitons and other shellfish contain mainly lutein, zeaxanthin, fucoxanthin, and their metabolites [160]. Mytiloxanthin is a characteristic carotenoid in marine mussels and oysters. Several sea snails, such as the triton *Charonia sauliae*, are carnivores preying upon starfish, and as a consequence, they accumulate astaxanthin and other typical carotenoids found in starfish (i.e., metabolites of β-carotene, diatoxanthin, and alloxanthin).

Crustaceans such as crabs, shrimps, and lobsters accumulate astaxanthin, which is synthesizedfrom β-carotene ingested from dietary algae [156,160]. In crustaceans, which can be purple, blue, or yellow pigmented, the color is provided by carotenoproteins (e.g., crustacyanin).

Echinoderms, including sea urchin, starfish and holoturians, contain caroteinoids such as β-carotene, echinenone, canthaxanthin, astaxanthin, fucoxanthin. Starfish are carnivorous mainly feeding on bivalves and small crustaceans thus accumulating mainly astaxanthin [166]. In sea urchins, carotenoids are important in egg production, development and biological functions [157]. In particular, β-echinenone is a major carotenoid found in the gonads of sea urchins (up to 50–60% of the total pigments), suggesting that it may have a role in their reproduction. Moreover, eggs of farmed sea urchin *Arbacia lixula* could represent a reliable source of astaxanthin and a convenient alternative to some microalgae, since a customized fodder can enhance the production of bioactive pigments from sea urchin gonads [167]. Previous studies revealed that the main pigments found in the gut wall of sea urchin *Paracentrotus lividus* were metabolic products of fucoxanthin (fucoxanthinol and amarouciaxanthin A) derived from the natural diet based on brown algae and diatoms. Lower levels of other dietary carotenoids (lutein and β-carotene) and other carotenoids not found in the diet (isozeaxanthin and echinenone, 20% total carotenoid) were also detected in the gut wall [168]. Novel marine carotenoids such as cucumariaxanthin A, B and C can be found in holothurians, such as *Cucumaria japonica* [156].

Tunicates, including ascidians, are filter feeders and contain mainly alloxanthin, fucoxanthin, mytiloxanthin, mytiloxanthinone and halocynthiaxanthin, which are potentially metabolic products of carotenoids assumed with dietary phytoplankton [169]. Peridinin and its metabolites are also found in tunicates. Two unique marine carotenoids, amarouciaxanthin A and B, were isolated from the ascidian *Amaroucium pliciferum* [169].

Several fish species accumulate carotenoids in an esterified form in their tegument and gonads. Salmonid fish and perciformes, which include 40% of all existent fish species, cannot synthesize astaxanthin from other carotenoids; therefore, they assume it from dietary crustacean zooplankton. This fish can convert astaxanthin to zeaxanthin. These fish accumulate astaxanthin in their muscles. Perciformes also contain tunaxanthin, which provides the bright yellow colour in the fins and skin of these fish [159]. 

Carotenoids, chemical structures and marine sources described in the Section 3 are summarized in the Table 2.

## 4. Industrial Applications of Carotenoids

### Carotenoids with High Market Value and New Opportunities and Challenges from Marine Organisms

Currently, most of the carotenoids for industrial use are produced through chemical synthesis, and only a small fraction is obtained by direct extraction from organisms, mainly of terrestrial origin [150]. However, the high demand by consumers for natural compounds and the global trend of the industry and market hint at a bright future for the employment of such natural molecules in functional cosmetics, pharmaceutical and nutraceutical sectors. The beneficial effects of carotenoids for human health described in this review can explain the increase of their extraction from marine sources and large-scale production. The global market for marine biotechnology products and processes is predicted to reach 4.8 billion USD by 2020, rising to 6.4 billion USD by 2025. In Europe, marine biotechnology was identified by the EU Blue Growth Strategy (2012) as an activity of high potential for the bioeconomy [170]. Ca. 1.20 billion USD is the estimated value for the global carotenoid market in 2016, which is expected to reach 1.53 billion USD by 2021, at a compound annual growth rate (CAGR) of 3.78% from 2016 to 2021 [171]. Such an economic growth related to these compounds is a direct result of the increased number of health conscious customers and a growing trend for natural products industries. The most successful carotenoids on the market are listed in Table 3, with industrially promising marine organisms. In particular, they are often used in aquaculture to reinforce fish color, which increases consumers’ perception of good quality and organoleptic properties of food, and in the case of bioactive carotenoids, for their nutraceutical and cosmeceutical properties.

Astaxanthin is one of the most successful carotenoids on the market, commonly extracted from industrial scale cultivation of the green microalgae *Haematococcus pluvialis*. Today, natural production of astaxanthin and other bioactive pigments is still considered more expensive than chemical synthesis, which remains the predominant production process because of its lower costs [171,172,173]. Many studies are investigating the best culture conditions to enhance the astaxanthin yield from *H. pluvialis*. To date, the green microalgae under high CO_2_ concentration (6%) and high light intensity (108 μmol photons m^−2^ s^−1^) conditions can produce about 50 mg/L of astaxanthin, which is double the amount of the specific carotenoid than the quantity produced by the wild-type strain [174].

Another successful carotenoid on the global market is β-carotene from its marine source *Dunaliella salina*. In the last decade, the global market was dominated by this microalgal species (together with Spirulina) [175]. *D. salina* is mostly used for the production of β-carotene. Indeed, under specific temperature and light intensity conditions (22 °C and 245.6 μmol m^−2^ s^−1^), 1.25 g/L of microalgal biomass can produce about 0.1 g/L of β-carotene [176,177]. The *D. salina* powder is industrially exploited as food colorant, but also for nutraceutical and health care products in many countries in the last 30 years [172,178]. However, the synthetic production of β-carotene still has a more competitive cost than natural β-carotene [179]. In fact, synthetic β-carotene has a price of ca. 300 €/kg, while the natural compound can reach more than double that cost [119]. From the quality point of view, natural β-carotene has better benefits on human health than the synthetic one. Indeed, the synthetic β-carotene contains only the trans isomer, which has lower liposolubility and antioxidant activity than the 9-cis isomer, which is found exclusively in the natural compounds together with the trans isomer [180].

The ketocarotenoid canthaxanthin is largely used as feed in salmon farming to guarantee the flesh color of fish. In recent years, the extremely halophilic microorganism *Haloferax alexandrines* has been investigated for its biotechnological potential in canthaxanthin large-scale production. This microorganism can grow at high NaCl concentrations (up to 25%), avoiding possible culture contaminations and high costs for asepsis conditions. One liter of *H. alexandrinus* culture can produce ca. 3 g of dried biomass, which contains 6.3 mg of total carotenoids; about 30% of the carotenoid pool is accounted by canthaxanthin [181]. In addition, when the *H. alexandrinus* culture is gradually diluted in lower NaCl concentration solutions it undergoes cell lysis. This represents another industrial advantage of *H. alexandrinus* culture, since cell lysis avoids the mechanical disruption step, thus allowing a cheaper and faster extraction procedure. The green microalgae *Chlorella zofingiensis* could be a valuable marine source for the production of carotenoids, especially canthaxanthin [126]. This microalga grown at 25 °C, with high light conditions (150 μmol photons m^−2^ s^−1^) exhibits up regulation of carotenogenic genes and an enhancement of the astaxanthin, zeaxanthin and canthaxanthin concentrations (118%, 430% and 178% higher than in the cultures before treatment with high light conditions, respectively). In addition, the thraustochytrid strain KH105 has been investigated as a potential industrial source of astaxanthin and canthaxanthin. This thraustochytrid strain, grown in a modified medium characterized by 10% glucose and 6% of nitrogen sources, can provide 6.1 mg of astaxanthin and more than 10 mg of canthaxanthin per liter of culture, thus suggesting its strong potential in the production of xanthophylls to be exploited in the food industry. The bacterium *Dietzia natronolimnaea* also found in deep-sea sediments has been investigated for the production of canthaxanthin [182]. The highest biomass yield (7.25 g/L) and canthaxanthin production (5.31 mg/L) was obtained when *D. natronolimnaea* HS-1 was subjected to a temperature of 31 °C, PH 7, without NaCl and with light (600 ± 50 lux). This yield is more than double that of *H. alexandrinus* (2.19 g of canthaxanthin per liter of culture). Other possible marine sources canthaxanthin of commercial/industrial interest are *H. pluvialis*, *Chlorella vulgaris* and *Coelastrella multistriata* [183,184,185].

So far, lutein is mainly extracted from terrestrial sources for industrial applications. In particular, the petals of marigold are the most exploited source (295 mg of carotenoid per 100 g of petals, 0.03% dried weight) [186]. This pigment is widely consumed as food colorant, but in the last decade, the production of nutraceutical formulations containing lutein has increased. Lutein is also one of the most representative bioactive pigments in marine microalgae *Murielopsis* sp. (0.6% dried weight) [187], *Chlorella protothecoides* (0.42% dried weight) [188] and *Scenedesmus almeriensis* (0.54% dried weight), which are largely studied for biotechnological applications. Lutein production from these cultures could be competitive with the lutein obtained from marigold, as high amounts of microalgal pigments (0.4–1.2% dry weight) can be obtained under controlled conditions [189].

Biosynthetic production from microorganism cultures is influenced by several factors, such as temperature, light, salinity and nutrient concentration. For instance, in *Dunaliella* sp. the high temperature (close to tolerance limit) can increase the production of lutein as well as β-carotene [123]. Levels of pH can influence differently lutein biosynthesis, depending on the type of microalgal culture system. In particular, at extreme pH values the batch cultivation of *D. salina* can increase lutein production, while continuous systems need optimum pH for the biosynthesis of high carotenoid amounts. Recent studies described the production of high lutein amounts (0.54% dry weight, 3.8 mg L^−1^ day^−1^) from the green microalga *Scenedesmus almeriensis* at 33 °C and 1625 μE/m^2^ s [190,191].

Oxidative factors are responsible of the increase in lutein production, since this carotenoid has a key role in the intracellular antioxidant defence system. Indeed, the presence of H_2_O_2_ and NaClO in the medium produce the alteration of redox balance in microalgae species, such as *Chlorella prothotecoides*, with higher production of lutein than in normal growth conditions [191]. For this reason, *C. prothotecoides* is considered one the most promising lutein-producing microalgae, since scientific advances have demonstrated a higher yield than from terrestrial sources.

Fucoxanthin, which is extracted from seaweeds such as *Laminaria japonica* and other species, is largely used as a base for food preparations and it is largely exploited as supplement for slimming diets, potentially due to its capability to increase basal metabolism. However, the content of fucoxanthin in marine macroalgae is not high enough (ca. 0.2 mg per g of fresh weight) for the large-scale production and, for this reason, they still need of more studies for efficient mariculture [192]. Conversely, microalgae have a greater potential than macroalgae for natural production of fucoxanthin. In particular, Kim and collaborators [193] studied the marine microalga *Isochrysis* aff. *Galbana* that showed a high content of fucoxanthin (18.3 mg per g of dried biomass), which can be extracted by using a cheap and efficient procedure. Moreover, fucoxanthin is the predominant carotenoid in the diatom *Phaeodactylum tricornutum* (15.7 mg per g of dry biomass) [194]. Another interesting microalga from the industrial point of view is the diatom *Odontella aurita*, which in a bubble column photobioreactor can produce 80 mg of fucoxanthin per L of culture (300 μmol photons m^−2^ s^−1^ and 18 mM of nitrate) demonstrating the feasibility of industrial scale-up of this species for the carotenoid production [195].

In summary, the exploitation of marine organisms for the industrial production of natural carotenoids is a fast growing sector, and due to its decreasing production costs and improvement of cultivation processes it is expected to become very competitive in the market for their higher biological value than synthetic products.

## 5. Conclusions

Marine biotechnology plays a crucial role in providing solutions for a sustainable and renewable food production, thus contributing to a wider bio-economy. New concepts of bioprocessing are emerging, utilizing close to 100% of the available biomass in sustainable multistream biorefineries. These processes deliver customized products, including the production of marine derived biomolecules, by the application of circular economy concepts to minimize waste and energy use [196]. In the last decade, the industrial production of carotenoids has become one of the most successful applications in the field of marine biotechnology. There is evidence, indeed, that the market demand for carotenoids from natural sources is increasing and there is a necessity to improve their production from natural and renewable sources as an alternative to more expensive and potentially non eco-friendly synthetic products [123]. To date, marine microalgae represent the principal source of marine carotenoids because they are renewable, eco-sustainable and at the same time better characterized for the production of these pigments than other marine organisms. However, the fast discovery rate of new marine (micro)-organisms even from previously unknown marine habitats paves the way to the identification of novel and sustainable molecules that are useful for human well-being. In particular, marine organisms such as fungi and prokaryotes have enormous potential for the development and marketing of new antioxidant compounds and arytenoids for different application fields [6].

## Figures and Tables

**Table 1:** In vitro and in vivo studies of biological roles of carotenoids.

Carotenoid	Effect	Model	Bioactive Concentration	Target	Reference
**Astaxanthin**	Antioxidant	Human monocytes (U-937)	10 μM	SHP-1	[30]
		Mice brain	2 mg/kg/day	MDA, NO, APOP, GSH.	[32]
		Leydig cells	10 μg/mL	StAR	[33]
	Antiproliferative	human prostatic adenocarcinoma (LNCaP)	10 μM	prostate specific antigen (PSA)	[86]
	immune system stimulation	transplantable methylcholanthrene-induced fibrosarcoma (Meth-A tumor)	40 mg/kg/day	interferon-g (IFN-γ)	[87]
	anti-obesity	Humans	0, 6, 12 and 18 mg/day	adiponectin	[103]
	Cardiovascular protective	spontaneously hypertensive rats (SHR)	50 mg/kg	blood pressure (BP)	[104]
**Fucoxanthin**	antioxidant and protective	Vero cells	5, 50, 100 and 200 µM (50 µM H_2_O_2_)	DNA	[35]
	UV protection	Human fibroblasts	5, 50 and 100 µM (50 mJ/cm^2^ UV-B)	DNA	[36]
	Antioxidant	Retinol deficiency rats	0.83 µM	CAT, GST and Na^+^K^+^ATPase activity	[37]
	Antiproliferative	leukemia cells (HD-60)	11.3 and 45.2 μM	DNA fragmentation	
		colorectal adenocarcinoma cells (Caco-2)	15.2 μM	DNA fragmentation	[65]
		colorectal adenocarcinoma cells (DLD-1)	15.2 μM	DNA fragmentation	[65]
		colorectal adenocarcinoma cells (CHT-29)	15.2 μM	DNA fragmentation	[65]
		human colorectal carcinoma (HCT116)	5 and 10 μM	Bcl-xL, PARP and caspase 3 and 7	[67,68,69,70]
		human prostate cancer (PC-3)	5 and 10 μM	Bcl-xL, PARP and caspase 3 and 7	[67,68,69,70]
		human urinary bladder cancer cells (EJ-1)	20 μM		[76]
	anti-obesity	Rats	2 mg	absorption of triglycerides , pancreatic lipase	[102]
**Fucoxanthinol**	Antiproliferative	human prostate cancer (PC-3)	2.0 μM	Bcl-xL, PARP and caspase 3 and 7	[67]
	anti-obesity	Rats	2 mg	absorption of triglycerides, pancreatic lipase	[102]
**Halocynthiaxanthin**	Antiproliferative	human neuroblastoma cells (GOTO)	5 μg/mL		[77]
**β-carotene**	Antioxidant	Smokers	20 mg	Breath pentane	[44]
	Cure of erythema	Humans	30 to 90 mg/day		[53]
	Antiproliferative	murine osteosarcoma (LM8)	30 µM		[79]
	Antiinfiammatory	human umbilical vein endothelial cells (HUVECs)	0.02 µmol/L	VCAM-1, ICAM-1 and E-Selectin	[96,98]
**Lutein**	ADM prevention	Human Dermal Lymphatic Endothelial Cells (HLEC)	5 µM	DNA, lipid and protein level	[56]
	Cardiovascular protective	Human monocytes	0.1, 1, 10 and 100 nM	LDL associated with artery wall	[106]
**Zeaxanthin**	ADM prevention	Human Dermal Lymphatic Endothelial Cells (HLEC)	5 µM	DNA, lipid and protein level	[56]
**Chantaxanthin**	Antiproliferative	human and murine melanoma (SK-MEL-2, JB/MS and B16F10)	10 μM		[80,81]
		fibrosarcoma cells (PYB6)	10 μM		[80,81]
	immune system stimulation	hamster buccal pouch carcinoma/macrophages	1.9 mg/mL	TNF-α	[83]
		T- and B-lymphocyte	2 g/kg		[84]
**Siphonaxanthin**	Antiproliferative	leukemia cells (HD-60)	20 μM	Bcl-2, caspase 3, GADD45α and DR5	[88]
**Lutein**	Antiinfiammatory	human umbilical vein endothelial cells (HUVECs)	0.5 µmol/L	VCAM-1, ICAM-1 and E-Selectin	[96,98]

**Table 2:** Carotenoids from marine organisms and molecular structures.

Carotenoid	Molecular Structure	Marine Sources
**Astaxanthin**	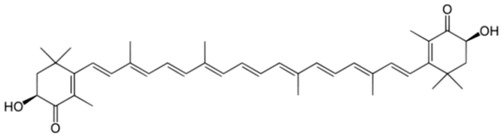	Chlorophyta: *Hematococcus pluvialis* Bacteria: *Agrobacterium* and *Paracoccus* genera Yeast: *Xanthophyllomyces*, *Rhodotorula* and *Phaffia* genera Fungi-like protists: *Thraustochytrium* strains ONC-T18 and CHN-1, Thraustochytriidae sp. AS4-A1 (*Ulkenia* sp.) and *Aurantiochytrium* sp. KH105 Sea snails: *Charonia sauliae* Sea Urchin: *Arbacia lixula* Found in: starfish, holoturians, crabs, shrimp, lobsters and shellfish
**Fucoxanthin**	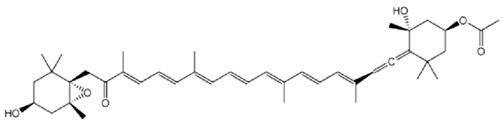	Seaweed: *Sargassum siliquastrum*, *Hijikia fusiformis*, *Undaria pinnatifida*, *Laminaria japonica*, *Alaria crassifolia*, *Cladosiphon okamuranus*, *Cystoseira hakodatensis*, *Eisenia bicyclis*, *Hijikia fusiformis*, *Ishige okamurae*, *Kjellmaniella crassifolia*, *Myagropsis myagroides*, *Padina tetrastromatica*, *Petalonia binghamiae* Diatoms: *Chaetoseros* sp., *Cylindrotheca closterium*, *Odontella aurita*, and *Phaeodactylum tricornutum* Found in: corals (due to their associations with cyanobacteria), sea urchin, starfish and holoturians
**β-Carotene**	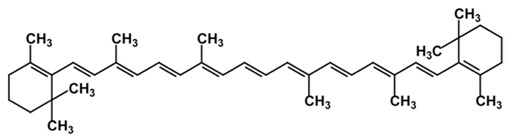	Microalga: *Dunaliella salina* Seagrass: *Posidonia oceanica*, *Cymodocea nodosa*, *Zostera noltii* and *Halophila stipulacea* Haloarchaea: *Haloferacaceae* family Fungi-like protists: *Thraustochytrium* strains ONC-T18 and CHN-1, Thraustochytriidae sp. AS4-A1 (*Ulkenia* sp.) and *Aurantiochytrium* sp. KH105 Found in: shellfish, cyanobacteria, sea urchin, starfish and holoturians
**Lutein**	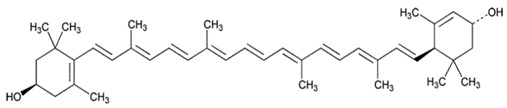	Microalga: *Dunaliella salina*, *Chlorella* sp Seagrass: *Posidonia oceanica*, *Cymodocea nodosa*, *Zostera noltii* and *Halophila stipulacea* Found in: corals (due to their associations with cyanobacteria) and shellfishes
**Mytiloxanthin**	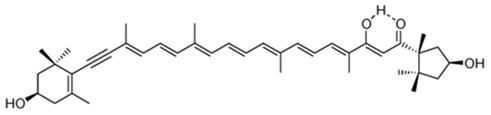	Found in: tunicates, mussels and oysters
**Zeaxanthin**	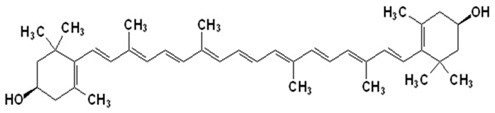	Microalga: *Dunaliella salina* Seagrass: *Posidonia oceanica*, *Cymodocea nodosa*, *Zostera noltii* and *Halophila stipulacea* Fungi-like protists: *Thraustochytrium* strains ONC-T18 and CHN-1, Thraustochytriidae sp. AS4-A1 (*Ulkenia* sp.) and *Aurantiochytrium* sp. KH105 Found in: corals (due to their associations with cyanobacteria) and shellfishes
**Saproxanthin**	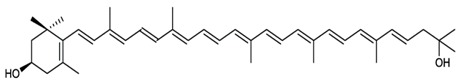	Found in: bacteria belonging to the family *Flavobacteriaceae*
**Myxol**	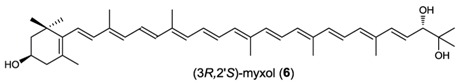	Found in: bacteria belonging to the family *Flavobacteriaceae*
**Lycopene**	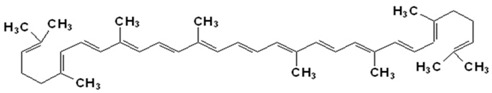	Found in: haloarchaea belonging to *Haloferacaceae* family
**Fucoxanthinol**	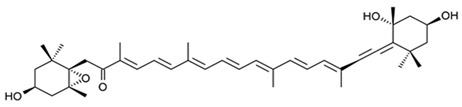	Found in: sea pineapple (*Halocynthia roretzi)* and sea urchin (*Paracentrotus lividus*)
**Halocynthiaxanthin**	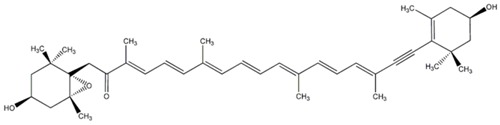	Found in: sea squirt and sea pineapple (e.g., *Halocynthia roretzi)*
**Canthaxanthin**	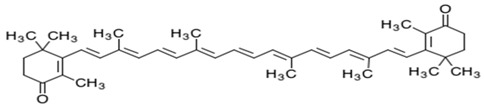	Fungi-like protists: *Thraustochytrium* strains ONC-T18 and CHN-1, Thraustochytriidae sp. AS4-A1 (*Ulkenia* sp*.*) and *Aurantiochytrium* sp. KH105 Found also in: sea urchin, starfish, holoturians, Crustaceans, algae and Cyanobacteria
**Peridinin**	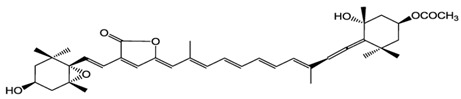	Microalgae: dinoflagellate *Heterocapsa triquetra* and dinoflagellates of the genus *Symbiodinium* Anemones: *Anemonia sulcata* Ascidian: *Amaroucium pliciferum*
**α-Carotene**	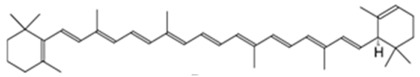	Microalga: *Dunaliella salina*
**Siphonaxanthin**	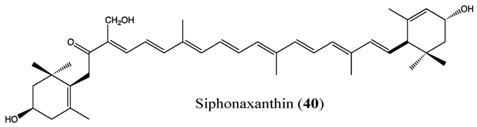	Found in: green algae
**Violaxanthin**	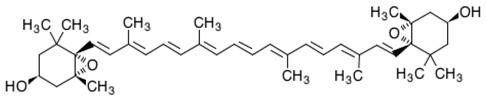	Seagrass: *Posidonia oceanica*, *Cymodocea nodosa*, *Zostera noltii* and *Halophila stipulacea*
**Antheraxanthin**	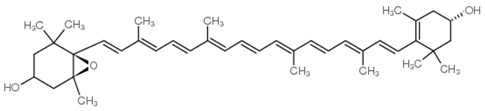	Seagrass: *Posidonia oceanica*, *Cymodocea nodosa*, *Zostera noltii* and *Halophila stipulacea*
**Neoxanthin**	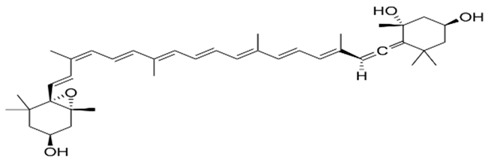	Seagrass: *Posidonia oceanica*, *Cymodocea nodosa*, *Zostera noltii* and *Halophila stipulacea*
**Bacterioruberin**	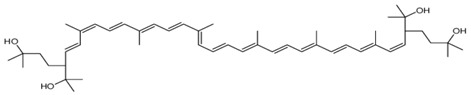	Found in : haloarchaea belonging to *Haloferacaceae* family (*Haloferax mediterranei)*
**β-Echinenone**	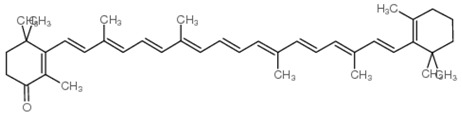	Found in: Gonads of sea urchin

**Table 3:** Main industrially produced carotenoids, their new potential marine sources and world manufacturing companies.

Carotenoids	Promising Marine Sources	Yield (by Scientific Studies)	Industrial Applications	Companies
Astaxanthin	*Haematococcus pluvialis*	50 mg/L	animal feed colorant, nutraceuticals, pharmaceuticals, cosmetics. (source: www.oilgae.com)	Alga Technologies (Israel), Cyanotech (USA), Jingzhou Natural Astaxanthin Inc. (China), Algaetech International (Malaysia), Parry Nutraceuticals (India), Mera Pharmaceuticals Inc. (USA), Fuji Chemicals (Sweden), Valensa International (USA).
	Thraustochytrid strain KH105	6.1 mg/L		
β-carotene	*Dunaliella salina*	100 mg/L	food and beverage colorant, food and feed additive, antioxidant agent, immune stimulating, antiaging, cancer and cardiovascular diseases preventive. (source: www.grandviewresearch.com)	AquaCarotene (USA), Cognis Nutrition & Health (Australia), Cyanotech (USA), Nikken Sohonsha Corporation (Japan), Tianjin Lantai Biotechnology (China), Parry Nutraceuticals (India), Seambiotic (Israel), Muradel (Australia).
Canthaxanthin	*Haloferax alexandrines*	2 mg/L	food and beverage colorant, pharmaceuticals, eggs yolk colorant, chicken skin colorant. (source: www.gminsights.com)	Hangzhou Spring Biotechnology Co., Ltd. (China), Xi’an Xin Sheng Bio-chem Co., Ltd. (China), Elitex Biological Technology (Autralia), Zipontchem Tech Co., Ltd. (China).
	Thraustochytrid strain KH105	10 mg/L		
	*Dietzia natronolimnaea* HS-1	7.25 mg/L		
Lutein	*Murielopsis* sp.	0.6% * d.w.	pharmaceuticals, dietary supplement, food, pet food, fish feed. (source: www.gminsights.com)	Orcas International, Inc. (USA), A Clover Nutrition (China), Reindeer Biotech (China), Ambe (India), Dynais (France), YBS Corporation (Japan), Aaco Vege-tech Company (China).
	*Chlorella protothecoides*	0.42% * d.w.		
	*Scenedesmus almeriensis*	0.54% * d.w. (3.8 mg/L)		
Fucoxanthin	*Laminalia japonica*	0.2 mg * f.w.	nutraceuticals, cosmeceuticals, pharmaceuticals.	AlgaNova International (China), Leili Natural Products Co., Ltd. (China).
	*Isochrysis* aff. *Galbana*	18.3 mg * d.w.		
	*Phaeodactylum tricornutum*	15.7 mg * d.w.		
	*Odontella aurita*	80 mg/L		

## References

[1] Britton G., Liaaen-Jensen S., Pfander H. (2004). Carotenoids Hand Book.

[2] Maoka T. (2011). Carotenoids in Marine Animals. Mar. Drugs.

[3] Nichols J.A., Katiyar S.K. (2010). Skin photoprotection by natural polyphenols: Anti-inflammatory, anti-oxidant and DNA repair mechanisms. Arch. Dermatol. Res..

[4] Berthon J.Y., Nachat-Kappes R., Bey M., Cadoret J.P., Renimel I., Filaire E. (2017). *Marine algae* as attractive source to skin care. Free Radic. Res..

[5] Gonzalez S., Gilaberte Y., Philips N., Juarranz A. (2011). Current Trends in Photoprotection—A New Generation of Oral Photoprotectors. Open Dermatol. J..

[6] Corinaldesi C., Barone G., Marcellini F., Dell’Anno A., Danovaro R. (2017). Marine Microbial-Derived Molecules and Their Potential Use in Cosmeceutical and Cosmetic Products. Mar. Drugs.

[7] Mata-Gómez L.C., Montañez J.C., Méndez-Zavala A., Aguilar C.N. (2014). Biotechnological production of carotenoids by yeasts: An overview. Microb. Cell Fact..

[8] Sy C., Dangles O., Borel P., Caris-Veyrat C. (2015). Interactions between Carotenoids from Marine Bacteria and Other Micronutrients: Impact on Stability and Antioxidant Activity. Mar. Drugs.

[9] Kim S.K. (2011). Marine Cosmeceuticals: Trends and Prospects.

[10] Aasen I.M., Ertesvåg H., Heggeset T.M.B., Liu B., Brautaset T., Vadstein O., Ellingsen T.E. (2016). Thraustochytrids as production organisms for docosahexaenoic acid (DHA), squalene, and carotenoids. Appl. Microbiol. Biotechnol..

[11] Ambati R.R., Phang S.M., Ravi S., Aswathanarayana R.G. (2014). Astaxanthin: Sources, extraction, stability, biological activities and its commercial applications—A review. Mar. Drugs.

[12] Craft B.D., Kerrihard A.L., Amarowicz R., Pegg R.B. (2012). Phenol-based antioxidants and the in vitro methods used for their assessment. Compr. Rev. Food Sci. Food. Saf..

[13] Lü J., Lin P.H., Yao Q., Chen C. (2010). Chemical and molecular mechanisms of antioxidants: Experimental approaches and model systems. J. Cell. Mol. Med..

[14] Hultqvust M., Olsson L.A., Gelderman K.A., Holmdah R. (2009). The protective role of ROS in autoimmune disease. Trends Immunol..

[15] Hughes G., Murphy M.P., Ledgerwood E.C. (2005). Mitochondrial reactive oxygen species regulate the temporal activation of nuclear factor κB to modulate tumor necrosis factor-induced apoptosis: Evidence from mitochondria-targeted antioxidants. Biochem. J..

[16] Storz P. (2005). Reactive oxygen species in tumor progression. Front. Biosci..

[17] Wiernsperger N.F. (2003). Oxidative stress as a therapeutic target in diabetes: Revisiting the controversy. Diabetes Metab..

[18] Halliwell B. (2007). Biochemistry of oxidative stress. Biochem. Soc. Trans..

[19] Pacher P., Beckman J.S., Liaudet L. (2007). Nitric oxide and peroxynitrite in health and disease. Physiol. Rev..

[20] Suzuki K. (2009). Anti-oxidants for therapeutic use: Why are only a few drugs in clinical use?. Adv. Drug Deliv. Rev..

[21] Wen X., Wu J., Wang F., Liu B., Huang C., Wei Y. (2013). Deconvoluting the role of reactive oxygen species and autophagy in human diseases. Free Radic. Biol. Med..

[22] Fiord J., Burda K. (2014). Potential role of carotenoids as antioxidant in human health and disease. Nutrients.

[23] Rodrigues E., Mariutti L.R.B., Mercadante A.Z. (2012). Scavenging Capacity of Marine Carotenoids against Reactive Oxygen and Nitrogen Species in a Membrane-Mimicking System. Mar. Drugs.

[24] Birben E., Sahiner U.M., Sackesen C., Erzurum S., Kalayci O. (2012). Oxidative stress and antioxidant defense. World Allergy Organ. J..

[25] D’Orazio N., Gemello E., Gammone M.A., de Girolamo M., Ficoneri C., Riccioni G. (2012). Fucoxantin: A treasure from the sea. Mar. Drugs.

[26] Gammone M.A., Riccioni G., D’Orazio N. (2015). Marine Carotenoids against Oxidative Stress: Effects on Human Health. Mar. Drugs.

[27] Guerin M., Huntley M.E., Olaizola M. (2003). *Haematococcus astaxanthin*: Applications for human health and nutrition. Trends Biotechnol..

[28] Wang S.L., He L.J., He T.B., Han W., Wang Q. (2015). Effect of astaxanthin on oxidative stress of red blood cells and peroxidation damage of membrane. Zhongguo Shi Yan Xue Ye Xue Za Zhi.

[29] Böhm F., Edge R., Truscott T.G. (2012). Interactions of dietary carotenoids with singlet oxygen (1O2) and free radicals: Potential effects for human health. Acta Biochim. Pol..

[30] Speranza L., Pesce M., Patruno A., Franceschelli S., DeLutiis M.A. (2012). Astaxanthin treatment reduced oxidative induced pro-inflammatory cytokinessecretion in U937: SHP-1 as a novel biological target. Mar. Drugs.

[31] Franceschelli S., Pesce M., Ferrone A., DeLutiis M.A., Patruno A., Grilli A., Felaco M., Speranza L. (2014). Astaxanthin Treatment Confers Protection against Oxidative Stress in U937 Cells Stimulated with Lipopolysaccharide Reducing O_2_^−^ Production. PLoS ONE.

[32] Al-Amin M.M., Akhter S., Hasan A.T., Alam T., Nageeb Hasan S.M., Saifullah A.R., Shohel C. (2015). The antioxidant effect of astaxanthin is higher in young mice than aged: A region specific study on brain. Metab. Brain Dis..

[33] Wang J.-Y., Lee Y.-J., Chou M.-C., Chang R., Chiu C.-H., Liang Y.-J., Wu L.-S. (2015). Astaxanthin Protects Steroidogenesis from Hydrogen Peroxide-Induced Oxidative Stress in Mouse Leydig Cells. Mar. Drugs.

[34] Rao A.V., Rao L.G. (2007). Carotenoids and human health. Pharmacol. Res..

[35] Heo S.J., Ko S.C., K S.M., Kang H.S., Kim J.P., Kim S.H., Lee K.W., Cho M.G., Jeon Y.J. (2008). Cytoprotective effect of fucoxanthin isolated from brown algae Sargassum siliquastrum against H_2_O_2_-induced cell damage. Eur. Food Res. Technol..

[36] Heo S.J., Jeon Y.J. (2009). Protective effect of fucoxanthin isolated from *Sargassum siliquastrum* on UV-B induced cell damage. J. Photochem. Photobiol. B Biol..

[37] Sangeetha R.K., Bhaskar N., Baskaran V. (2009). Comparative effects of β-carotene and fucoxanthin on retinol deficiency induced oxidative stress in rats. Mol. Cell. Biochem..

[38] Maoka T., Nishino A., Yasui H., Yamano Y., Wada A. (2016). Anti-Oxidative Activity of Mytiloxanthin, a Metabolite of Fucoxanthin in Shellfish and Tunicates. Mar. Drugs.

[39] Hirayama O., Nakamura K., Hamada S., Kobayashi K. (1994). Singlet oxygen quenching ability of naturally occurring carotenoids. Lipids.

[40] Shimidzu N., Goto M., Miki W. (1996). Carotenoids as singlet oxygen quenchers in marine organism. Fish. Sci..

[41] Giblin F.J. (2000). Glutathione: A vital lens antioxidant. J. Ocul. Pharmacol. Ther..

[42] Shindo K., Kimura M., Iga M. (2004). Potent antioxidative activity of cacalol, a sesquiterpene contained in *Cacalia delphiniifolia* Sleb et Zucc. Biosci. Biotechnol. Biochem..

[43] Shindo K., Kikuta K., Suzuki A., Katsuta A., Kasai H., Yasumoto-Hirose M., Matsuo Y., Takaichi S. (2007). Rare carotenoids, (3R)-saproxanthin and (3R,2′S)-myxol, isolated from novel marine bacteria (*Flavobacteriaceae*) and their antioxidative activities. Appl. Microbiol. Biotechnol..

[44] Allard J.P., Royall D., Kurian R., Muggli R., Jeejeebhoy K.N. (1994). Effects of beta-carotene supplementation on lipid peroxidation in humans. Am. J. Clin. Nutr..

[45] Amengual J., Lobo G.P., Golczak M., Li H.N., Klimova T., Hoppel C.L., Wyss A. (2011). A mitochondrial enzyme degrades carotenoids and protects against oxidative stress. FASEB J..

[46] Sies H., Stahl W. (1995). Vitamins E and C, beta-carotene, and other carotenoids as antioxidants. Am. J. Clin. Nutr..

[47] Younga A.J., Lowe G.M. (2001). Antioxidant and Prooxidant Properties of Carotenoids. Arch. Biochem. Biophys..

[48] Ranadive N.S., Menon I.A., Shirwadkar S., Persad S.D. (1989). Quantitation of cutaneous inflammation induced by reactive species generated by UV-visible irradiation of rose bengal. Inflammation.

[49] Stahl W., Sies H. (2005). Bioactivity and protective effects of natural carotenoids. Biochim. Biophys. Acta.

[50] Böhm F., Edge R., Lange L., Truscott T.G. (1998). Enhanced protection of human cells against ultraviolet light by antioxidant combinations involving dietary carotenoids. J. Photochem. Photobiol..

[51] Böhm F., Edge R., McGarvey D.J., Truscott T.G. (1998). Beta-carotene with vitamins E and C offers synergistic cell protection against NO_x_. FEBS Lett..

[52] Sansone C., Galasso C., Orefice I., Nuzzo G., Luongo E., Cutignano A., Romano G., Brunet C., Fontana A., Esposito F. (2017). The green microalga *Tetraselmis suecica* reduces oxidative stress and induces repairing mechanisms in human cells. Sci. Rep..

[53] Stahl W., Sies H. (2012). β-Carotene and other carotenoids in protection from sunlight. Am. J. Clin. Nutr..

[54] Koepcke W., Krutmann J. (2008). Protection from sunburn with β-carotene—A meta-analysis. Photochem. Photobiol..

[55] Darvin M.E., Sterry W., Lademann J., Vergou T. (2011). The role of carotenoids in human skin. Molecules.

[56] Gao S., Qin T., Liu Z., Caceres M.A., Ronchi C.F., Chen C.-Y.O., Shang F. (2011). Lutein and zeaxanthin supplementation reduces H_2_O_2_-induced oxidative damage in human lens epithelial cells. Mol. Vis..

[57] Hammond B.R., Johnson B.A., George E.R. (2014). Oxidative photodegradation of ocular tissues: Beneficial effects of filtering and exogenous antioxidants. Exp. Eye Res..

[58] Olmedilla-Alonso B., Beltrán-de-Miguel B., Estévez-Santiago R., Cuadrado-Vives C. (2014). Markers of lutein and zeaxanthin status in two age groups of men and women: Dietary intake, serum concentrations, lipid profile and macular pigment optical density. Nutr. J..

[59] Wu J., Cho E., Willett W.C., Sastry S.M., Schaumberg D.A. (2015). Intakes of lutein, zeaxanthin, and other carotenoids and age-related macular degeneration during 2 decades of prospective follow-up. JAMA Ophthalmol..

[60] Nishino H., Murakoshi M., Ii T., Takemura M., Kuchide M., Kanazawa M., Mou X.Y., Wada S., Masuda M., Ohsaka Y. (2002). Carotenoids in Cancer Chemoprevention. Cancer Metastasis Rev..

[61] Rock C.L., Britton G., Liaaen-Jensen S., Pfander H. (2009). Carotenoids and Cancer. Carotenoids.

[62] Das A., Yoon S.H., Lee S.H., Kim J.Y., Oh D.K., Kim S.W. (2007). An update on microbial carotenoid production: Application of recent metabolic engineering tools. Appl. Microbiol. Biotechnol..

[63] Dembitsky V.M., Maoka T. (2007). Allenic and cumulenic lipids. Prog. Lipid Res..

[64] Mori K., Ooi T., Hiraoka M., Oka N., Hamada H., Tamura M., Kusumi T. (2004). Fucoxanthin and its metabolites in edible brown algae cultivated in deep seawater. Mar. Drugs.

[65] Hosokawa M., Wanezaki S., Miyauchi K., Kurihara H., Kohno H., Kawabata J., Takahashi K. (1999). Apoptosis-inducing effect of fucoxanthin on human leukemia cell HL-60. Food Sci. Technol. Res..

[66] Hosokawa M., Kudo M., Maeda H., Kohno H., Tanaka T., Miyashita K. (2004). Fucoxanthin induces apoptosis and enhances the antiproliferative effect of the PPARgamma ligand, troglitazone, on colon cancer cells. Biochim. Biophys. Acta.

[67] Kotake-Nara E., Asai A., Nagao A. (2005). Neoxanthin and fucoxanthin induce apoptosis in PC-3 human prostate cancer cells. Cancer Lett..

[68] Hoang V.C., Jong-Bang E. (2015). Marine carotenoids: Bioactivities and potential benefits to human health. Crit. Rev. Food Sci. Nutr..

[69] Kotake-Nara E., Sugawara T., Nagao A. (2005). Antiproliferative effect of neoxanthin and fucoxanthin on cultured cells. Fish. Sci..

[70] Kim K.N., Heo S.J., Kang S.M., Ahn G., Jeon Y.J. (2010). Fucoxanthin induces apoptosis in human leukemia HL-60 cells through a ROS-mediated Bcl-xL pathway. Toxicol. In Vitro.

[71] Das S.K., Hashimoto T., Shimizu K., Yoshida T., Sakai T., Sowa Y., Komoto A., Kanazawa K. (2005). Fucoxanthin induces cell cycle arrest at G0/G1 phase in human colon carcinoma cells through up-regulation of p21WAF1/Cip1. Biochim. Biophys. Acta.

[72] Yu R.X., Hu X.M., Xu S.Q., Jiang Z.J., Yang W. (2011). Effects of fucoxanthin on proliferation and apoptosis in human gastric adenocarcinoma MGC-803 cells via JAK/STAT signal pathway. Eur. J. Pharmacol..

[73] Satomi Y., Nishino H. (2009). Implication of mitogen-activated protein kinase in the induction of G1 cell cycle arrest and gadd45 expression by the carotenoid fucoxanthin in human cancer cells. Biochim. Biophys. Acta.

[74] Satomi Y. (2012). Fucoxanthin induces GADD45A expression and G1 arrest with SAPK/JNK ctivation in LNCap human prostate cancer cells. Anticancer Res..

[75] Kotake-Nara E., Kushiro M., Zhang H., Sugawara T., Miyashita K., Nagao A. (2001). Carotenoids affect proliferation of human prostate cancer cells. J. Nutr..

[76] Zhang Z., Zhang P., Hamada M., Takahashi S., Xing G., Liu J., Sugiura N. (2008). Potential chemoprevention effect of dietary fucoxanthin on urinary bladder cancer EJ-1 cell line. Oncol. Rep..

[77] Nishino H., Tsushima M., Matsuno T., Tanaka Y., Okuzumi J., Murakoshi M. (1992). Anti-neoplastic effect of halocynthiaxanthin, a metabolite of fucoxanthin. Anticancer Drugs.

[78] Konishi I., Hosokawa M., Sashima T., Kobayashi H., Miyashita K. (2006). *Halocynthiaxanthin* and fucoxanthinol isolated from *Halocynthiaroretzi* induce apoptosis in human leukemia, breast and colon cancer cells. Comp. Biochem. Physiol. Part C Toxicol. Pharmacol..

[79] Rokkaku T., Kimura R., Ishikawa C., Yasumoto T., Senba M., Kanaya F., Mori N. (2013). Anticancer effects of marine carotenoids, fucoxanthin and its deacetylated product, fucoxanthinol, on osteosarcoma. Int. J. Oncol..

[80] Huang D.S., Odeleye O.E., Watson R.R. (1992). Inhibitory effects of canthaxanthin on in vitro growth of murine tumor cells. Cancer Lett..

[81] Palozza P., Maggiano N., Calviello G., Lanza P., Piccioni E., Ranelletti F.O., Bartoli G.M. (1998). Canthaxanthin induces apoptosis in human cancer cell lines. Carcinogenesis.

[82] Abdel-Fatth G., Watzl B., Huang D., Watson R.R. (1993). Betacarotene in vitro stimulates tumor necrosis factor alpha and interleukin 1 alpha secretion by human peripheral blood mononuclear cells. Nutr. Res..

[83] Shklar G., Schwartz J. (1988). Tumor necrosis factor in experimental cancer regression with alphatocopherol, beta-carotene, canthaxanthin and algae extract. Eur. J. Cancer Clin. Oncol..

[84] Bendich A., Shapiro S.S. (1986). Effect of beta carotene and canthaxanthin on the immune response in rats. J. Nutr..

[85] Sugawara T., Yamashita K., Sakai S., Asai A., Nagao A., Shiraishi T., Hirata T. (2007). Induction of apoptosis in DLD-1 human colon cancer cells by peridinin isolated from the dinoflagellate, *Heterocapsa triquetra*. Biosci. Biotechnol. Biochem..

[86] Sharoni Y., Agemy L., Giat U., Kirilov E., Danilenko M., Levy J. Lycopene and astaxanthin inhibit human prostate cancer cell proliferation induced by androgens. Proceedings of the 13th International Symposium on Carotenoids.

[87] Jyonouchi H., Sun S., Iijima K., Gross M.D. (2000). Antitumoractivity of astaxanthin and its mode of action. Nutr. Cancer.

[88] Ganesan P., Noda K., Manabe Y., Ohkubo T., Tanaka Y., Maoka T., Sugawara T., Hirata T. (2011). Siphonaxanthin, a marine algal carotenoids from green algae, effectively induces apoptosis in humanleukemia (HL-60) cells. Biochim. Biophys. Acta.

[89] Chan J.M., Gann P.H., Giovannucci E.L. (2005). Role of diet in prostate cancer development and progression. J. Clin. Oncol..

[90] Wertz K., Siler U., Góralczyk R. (2004). Lycopene: Modes of action to promote prostate health. Arch. Biochem. Biophys..

[91] Chaiter Y., Gruber S.B., Ben-Amotz A., Almog R., Rennert H.S., Fischler R., Rozen G., Rennert G. (2009). Smoking attenuates the negative association between carotenoids consumption and colorectal cancer risk. Cancer Causes Control.

[92] Jeon Y.J., Myun S.K., Lee E.H., Kim Y., Chang Y.J., Ju W., Cho H.J., Seo H.G., Huh B.Y. (2011). Effects of beta-carotene supplements on cancer prevention: Meta-analysis of randomized controlled trials. Nutr. Cancer.

[93] Góralczyk R. (2009). β-Carotene and lung cancer in smokers: Review of hypotheses and status of research. Nutr. Cancer.

[94] Vogiatzi G., Tousoulis D., Stefanadis C. (2009). The role of oxidative stress in atherosclerosis. Hell. J. Cardiol..

[95] Gori T., Nzel T.M. (2011). Oxidative stress and endothelial dysfunction: Therapeutic implications. Ann. Med..

[96] Di Pietro N., Di Tonno P., Pandolfi A. (2016). Carotenoids in cardiovascular disease prevenction. JSM Atheroscler..

[97] García-Fernández E., Rico-Cabanas L., Rosgaard N., Estruch R., Bach-Faig A. (2014). Mediterranean diet and cardiodiabesity: A review. Nutrients.

[98] Di Tomo P., Canali R., Ciavardelli D., Di Silvestre S., De Marco A., Giardinelli A., Pipino C., Di Pietro N., Virgili F., Pandolfi A. (2012). β-Carotene and lycopene affect endothelial response to TNF-a reducing nitro-oxidative stress and interaction with monocytes. Mol. Nutr. Food Res..

[99] Lee D.K., Grantham R.N., Mannion J.D., Trachte A.L. (2006). Carotenoids enhance phosphorylation of Akt and suppress tissue factor activity in human endothelial cells. J. Nutr. Biochem..

[100] Monroy-Ruiz J., Sevilla M.A., Carron R., Montero M.J. (2011). Astaxanthin-enriched-diet reduces blood pressure and improves cardiovascular parameters in spontaneously hypertensive rats. Pharmacol. Res..

[101] George T.W., Paterson E., Waroonphan S., Gordon M.H., Lovegrove J.A. (2012). Effects of chronic consumption of fruit and vegetable puree-based drinks on vasodilation, plasma oxidative stability and antioxidant status. J. Hum. Nutr. Diet..

[102] Matsumoto M., Hosokawa M., Matsukawa N., Hagio M., Shinoki A., Nishimukai M., Hara H. (2010). Suppressive effects of the marine carotenoids, fucoxanthin and fucoxanthinol on triglyceride absorption in lymph duct-cannulated rats. Eur. J. Nutr..

[103] Yoshida H., Yanai H., Ito K., Tomono Y., Koikeda T., Tsukahara H., Tada N. (2010). Administration of natural astaxanthin increases serum HDL-cholesterol and adiponectin in subjects with mild hyperlipidemia. Atherosclerosis.

[104] Hussein G., Nakamura M., Zhao Q., Iguchi T., Goto H., Sankawa U., Watanabe H. (2005). Antihypertensive and neuroprotective effects of astaxanthin in experimental animals. Biol. Pharm. Bull..

[105] Iwamoto T., Hosoda K., Hirano R., Kurata H., Matsumoto A., Miki W., Kondo K. (2000). Inhibition of low-density lipoprotein oxidation by astaxanthin. J. Atheroscler. Thromb..

[106] Dwyer J.H., Navab M., Dwyer K.M., Hassan K., Sun P., Shircore A., Hama-Levy S., Hough G., Wang X., Drake T. (2001). Oxygenated carotenoid lutein and progression of early atherosclerosis: The Los Angeles atherosclerosis study. Circulation.

[107] The Alpha-Tocopherol, Beta-Carotene Cancer Prevention Study Group (1994). The effect of vitamin E and beta-carotene on the incidence of lung cancer and other cancers in male smokers. N. Engl. J. Med..

[108] Tornwall M.E., Virtamo J., Korhonen P.A. (2004). Effect of α-tocopherol and β-carotene supplementation on coronary heart disease during the 6-year post-trial follow-up in the ATBC study. Eur. Heart J..

[109] Hennekens C.H., Buring J.E., Manson J.E., Stampfer M.J., Rosner B., Cook N.R., Belanger C., LaMotte F., Gaziano J.M., Ridker P.M. (1996). Lack of effect of long-term supplementation with beta carotene on the incidence of malignant neoplasms and cardiovascular disease. N. Engl. J. Med..

[110] Heart Protection Study Collaborative Group (2002). MRC/BHF Heart Protection Study of antioxidant vitamin supplementation in 20,536 high-risk individuals: A randomised placebo-controlled trial. Lancet.

[111] Maiani G., Castón M.J., Catasta G., Toti E., Cambrodón I.G., Bysted A., Granado-Lorencio F., Olmedilla-Alonso B., Knuthsen P., Valoti M. (2009). Carotenoids: Actual knowledge on food sources, intakes, stability and bioavailability and their protective role in humans. Mol. Nutr. Food Res..

[112] Yonekura L., Nagao A. (2007). Intestinal absorption of dietary carotenoids. Mol. Nutr. Food Res..

[113] Suleria H.A.R., Osborne S., Masci P., Gobe G. (2015). Marine-Based Nutraceuticals: An Innovative Trend in the Food and Supplement Industries. Mar. Drugs.

[114] Britton G., Britton G., Liaaen-Jensen S., Pfander H. (2012). Carotenoids.

[115] Serive B., Nicolau E., Bérard J.B., Kaas R., Pasquet V., Picot L., Cadoret J.P. (2017). Community analysis of pigment patterns from 37 microalgae strains reveals new carotenoids and porphyrins characteristic of distinct strains and taxonomic groups. PLoS ONE.

[116] Cadoret J.P., Garnier M., Saint-Jean B. (2012). Microalgae, functional genomics and biotechnology. Adv. Botanic. Res..

[117] Hamed I. (2016). The Evolution and Versatility of Microalgal Biotechnology: A Review. Compr. Rev. Food Sci. Food Saf..

[118] Martins A., Vieira H., Gaspar H., Santos S. (2014). Marketed Marine Natural Products in the Pharmaceutical and Cosmeceutical Industries: Tips for Success. Mar. Drugs.

[119] Guedes A.C., Amaro H.M., Malcata F.X. (2011). Microalgae as sources of carotenoids. Mar. Drugs.

[120] Raposo M.F.D.J., de Morais A.M.M.B., de Morais R.M.S.C. (2015). Carotenoids from marine microalgae: A valuable natural source for the prevention of chronic diseases. Mar. Drugs.

[121] Pulz O., Gross W. (2004). Valuable products from biotechnology of microalgae. Appl. Microbiol. Biotechnol..

[122] Mulders K.J., Lamers P.P., Martens D.E., Wijffels R.H. (2014). Phototrophic pigment production with microalgae: Biological constraints and opportunities. J. Phycol..

[123] García-González M., Moreno J., Manzano J.C., Florencio F.J., Guerrero M.G. (2005). Production of Dunaliella salina biomass rich in 9-cis-β-carotene and lutein in a closed tubular photobioreactor. J. Biotechnol..

[124] Lamers P.P., Janssen M., De Vos R.C., Bino R.J., Wijffels R.H. (2008). Exploring and exploiting carotenoid accumulation in Dunaliella salina for cell-factory applications. Trends Biotechnol..

[125] Kovač1 D.J., Simeunović J.B., Babić O.B., Mišan2 A.Č., Milovanović I.L. (2014). Algae in food and feed. Food Feed Res..

[126] Liu J., Sun Z., Gerken H., Liu Z., Jiang Y., Chen F. (2014). Chlorella zofingiensis as an alternative microalgal producer of astaxanthin: Biology and industrial potential. Mar. Drugs.

[127] Cerón-García M.D.C., Campos-Pérez I., Macías-Sánchez M.D., Bermejo-Román R., Fernández-Sevilla J.M., Molina-Grima E. (2010). Stability of carotenoids in Scenedesmus almeriensis biomass and extracts under various storage conditions. J. Agric. Food Chem..

[128] Macías-Sánchez M.D., Fernandez-Sevilla J.M., Fernández F.A., García M.C., Grima E.M. (2010). Supercritical fluid extraction of carotenoids from Scenedesmus almeriensis. Food Chem..

[129] Sun Z., Li T., Zhou Z.G., Jiang Y. (2015). Microalgae as a source of lutein: Chemistry, biosynthesis, and carotenogenesis. Adv. Biochem. Eng. Biotechnol..

[130] Stange C. (2016). Carotenoids in Nature: Biosynthesis, Regulation and Function.

[131] Kobayashi M., Kurimura Y., Tsuji Y. (1997). Light-independent, astaxanthin production by the green microalga *Haematococcus pluvialis* under salt stress. Biotechnol. Lett..

[132] León R., Couso I., Fernández E. (2007). Metabolic engineering of ketocarotenoids biosynthesis in the unicelullar microalga *Chlamydomonas reinhardtii*. J. Biotechnol..

[133] Takaichi S. (2011). Carotenoids in Algae: Distributions, Biosyntheses and Functions. Mar. Drugs.

[134] Yan X., Chuda Y., Suzuki M., Nagata T. (1999). Fucoxanthin as the major antioxidant in *Hijikia fusiformis*, a common edible seaweed. Biosci. Biotechnol. Biochem..

[135] Piovan A., Seraglia R., Bresin B., Caniato R., Filippini R. (2013). Fucoxanthin from *Undaria pinnatifida*: Photostability and coextractive effects. Molecules.

[136] Noviendri D., Salleh H.M., Taher M., Miyashita K., Ramli N. (2011). Fucoxanthin extraction and fatty acid analysis of Sargassum binderi and *S. duplicatum*. J. Med. Plant Res..

[137] Wang W.J., Wang G.C., Zhang M., Tseng C.K. (2005). Isolation of fucoxanthin from the rhizoid of *Laminaria japonica* Aresch. J. Integr. Plant Biol..

[138] Zaragozá M.C., López D., Sáiz M.P., Poquet M., Pérez J., Puig-Parellada P., Burtin P. (2008). Toxicity and antioxidant activity in vitro and in vivo of two *Fucus vesiculosus* extracts. J. Agric. Food Chem..

[139] Peng J., Yuan J.-P., Wu C.-F., Wang J.-H. (2011). Fucoxanthin, a Marine Carotenoid Present in Brown Seaweeds and Diatoms: Metabolism and Bioactivities Relevant to Human Health. Mar. Drugs.

[140] Short F., Carruthers T., Dennison W., Waycott M. (2007). Global seagrass distribution and diversity: A bioregional model. J. Exp. Mar. Biol. Ecol..

[141] Hemminga M.A., Duarte C.M. (2000). Seagrass Ecology.

[142] Casazza G., Mazzella L. (2002). Photosynthetic pigment composition of marine angiosperms: Preliminary characterization of mediterranean seagrasses. Bull. Mar. Sci..

[143] Silva J., Barrote I., Costa M.M., Albano S., Santos R. (2013). Physiological responses of *Zostera marina* and *Cymodocea nodosa* to light-limitation stress. PLoS ONE.

[144] Chowdhury G.R., Agarwal S., Mitra A. (2017). Astaxanthin Pattern in Mangroves: A Case of Species-Specificity. Biomed. J. Sci. Tech. Res..

[145] Vílchez C., Forján E., Cuaresma M., Bédmar F., Garbayo I., Vega J.M. (2011). Marine Carotenoids: Biological Functions and Commercial Applications. Mar. Drugs.

[146] Miyashita K. (2009). Function of marine carotenoids. Forum. Nutr..

[147] Rodrigo-Baños M., Garbayo I., Vílchez C., Bonete M.J., Martínez-Espinosa R.M. (2015). Carotenoids from Haloarchaea and their potential in biotechnology. Mar. Drugs.

[148] Chen C.W., Hsu S.H., Lin M.T., Hsu Y.H. (2015). Mass production of C50 carotenoids by *Haloferax mediterranei* in using extruded rice bran and starch under optimal conductivity of brined medium. Bioproc. Biosyst. Eng..

[149] Zakar T., Laczko-Dobos H., Toth T.N., Gombos Z. (2016). Carotenoids assist in cyanobacterial Photosystem II assembly and function. Front. Plant Sci..

[150] Mezzomo N., Ferreira S.R. (2016). Carotenoids functionality, sources, and processing by supercritical technology: A review. J. Chem..

[151] Bumbak F., Cook S., Zachleder V., Hauser S., Kovar K. (2011). Best practices in heterotrophic high-cell-density microalgal processes: Achievements, potential and possible limitations. Appl. Microbiol. Biotechnol..

[152] Raghukumar S. (2002). Ecology of the marine protists, the *Labyrinthulomycetes* (*Thraustochytrids* and *Labyrinthulids*). Eur. J. Protistol..

[153] Suen Y.L., Tang H., Huang J., Chen F. (2014). Enhanced production of fatty acids and astaxanthin in Aurantiochytrium sp. by the expression of *Vitreoscilla hemoglobin*. J. Agric. Food Chem..

[154] Cardoso L.A., Karp S.G., Vendruscolo F., Kanno K.Y., Zoz L.I., Carvalho J.C. (2017). Biotechnological Production of Carotenoids and Their Applications in Food and Pharmaceutical Products. Carotenoids.

[155] Tsushima M., Matsuno T. (1990). Comparative biochemical studies of carotenoids in sea-urchins—I. Comp. Biochem. Physiol. Part B Comp. Biochem..

[156] Matsuno T. (2001). Aquatic animal carotenoids. Fish. Sci..

[157] Tsushima M. (2007). Carotenoids in sea urchins. Dev. Aquac. Fish. Sci..

[158] Liaaen-Jensen S., Renstrøm B., Ramdahl T., Hallenstvet M., Bergquist P. (1982). Carotenoids of marine sponges. Biochem. Syst. Ecol..

[159] Maoka T., Mochida K., Kozuka M., Ito Y., Fujiwara Y., Hashimoto K., Nishino H. (2001). Cancer chemopreventive activity of carotenoids in the fruits of red paprika *Capsicum annuum* L.. Cancer Lett..

[160] Maoka T., Akimoto N., Tsushima M., Komemushi S., Mezaki T., Iwase F., Takahashi Y., Sameshima N., Mori M., Sakagami Y. (2011). Carotenoids in Marine Invertebrates Living along the Kuroshio Current Coast. Mar. Drugs.

[161] Osinga R., Schutter M., Griffioen B., Wijffels R.H., Verreth J.A., Shafir S., Lavorano S. (2011). The biology and economics of coral growth. Mar. Biotechnol..

[162] Apprill A.M., Gates R.D. (2007). Recognizing diversity in coral symbiotic dinoflagellate communities. Mol. Ecol..

[163] Daigo K., Nakano Y., Casareto B.E., Suzuki Y., Shioi Y. High-performance liquid chromatographic analysis of photosynthetic pigments in corals: An existence of a variety of epizoic, endozoic and endolithic algae. Proceedings of the 11th International Coral Reef Symposium.

[164] Venn A.A., Wilson M.A., Trapido-Rosenthal H.G., Keely B.J., Douglas A.E. (2006). The impact of coral bleaching on the pigment profile of the symbiotic alga, *Symbiodinium*. Plant Cell Environ..

[165] Bjerkeng B. (2008). Carotenoids in aquaculture: Fish and crustaceans. Carotenoids.

[166] Matsuno T., Krinsky N.I., Mathews-Roth M.M., Taylor R.F. (1989). Animal carotenoids. Carotenoids Chemistry and Biology.

[167] Cirino P., Brunet C., Ciaravolo M., Galasso C., Musco L., Vega Fernández T., Sansone C., Toscano A. (2017). The sea urchin *Arbacia lixula*: A novel natural source of astaxanthin. Mar. Drugs.

[168] Symonds R.C., Kelly M.S., Caris-Veyrat C., Young A.J. (2007). Carotenoids in the sea urchin *Paracentrotus lividus*: Occurrence of 9′-cis-echinenone as the dominant carotenoid in gonad colour determination. Comp. Biochem. Physiol. Part B Biochem. Mol. Biol..

[169] Matsuno T., Ookubo M., Komori T. (1985). Carotenoids of tunicates, III. The structural elucidation of two new marine carotenoids, amarouciaxanthin A and B. J. Nat. Prod..

[170] European Marine Board and Marine Biotechnology (ERANET) (2017). Marine Biotechnology: Advancing Innovation in Europe’s Bioeconomy. EMB Policy Brief.

[171] Markets and Markets Website—New Market Reports. http://www.marketsandmarkets.com/search.asp?Search=carotenoid&x=0&y=0.

[172] Misawa N. (2009). Pathway engineering of plants toward astaxanthin production. Plant Biotechnol..

[173] Raja R., Hemaiswarya S., Kumar N.A., Sridhar S., Rengasamy R. (2008). A perspective on the biotechnological potential of Microalgae. Crit. Rev. Microbiol..

[174] Cheng J., Li K., Yang Z., Zhou J., Cen K. (2016). Enhancing the growth rate and astaxanthin yield of *Haematococcus pluvialis* by nuclear irradiation and high concentration of carbon dioxide stress. Bioresour. Technol..

[175] Spolaore P., Joannis-Cassan C., Duran E., Isambert A. (2006). Commercial applications of Microalgae. J. Biosci. Bioeng..

[176] Hosseini Tafreshi A., Shariati M. (2009). Dunaliella biotechnology: Methods and applications. J. Appl. Microbiol..

[177] Wu Z., Duangmanee P., Zhao P., Juntawong N., Ma C. (2016). The Effects of Light, Temperature, and Nutrition on Growth and Pigment Accumulation of Three *Dunaliella salina* Strains Isolated from Saline Soil. Jundishapur J. Microbiol..

[178] Guedes A.C., Amaro H.M., Malcata F.X. (2011). Microalgae as sources of high added-value compounds? A brief review of recent work. Biotechnol. Prog..

[179] Jaswir I., Noviendri D., Hasrini R.F., Octavianti F. (2011). Carotenoids: Sources, medicinal properties and their application in food and nutraceutical industry. J. Med. Plants Res..

[180] Singh O.V. (2017). Bio-Pigmentation and Biotechnological Implementations.

[181] Asker A., Ohta Y. (2002). Production of canthaxanthin by *Haloferax alexandrinus* under non-aseptic conditions and a simple, rapid method for its extraction. Appl. Microbiol. Biotechnol..

[182] Khodaiyan F., Razavi S.H., Emam-Djomeh Z., Mousavi S.M., Hejazi M.A. (2007). Effect of culture conditions on canthaxanthin production by *Dietzia natronolimnaea* HS-1. J. Microbiol. Biotechnol..

[183] Demming-Adams B., Adams W.W. (2002). Antioxidants in photosynthesis and human nutrition. Science.

[184] El-Baky H.H.A., El-Baz F.K., El-Baroty G.S. (2003). Spirulinaspecies as a source of carotenoids and α-tocopherol and its anticarcinoma factors. Biotechnology.

[185] Abe K., Hattor H., Hiran M. (2005). Accumulation and antioxidant activity of secondary carotenoids in the aerial microalga *Coelastrella striolata* var. *multistriata*. Food Chem..

[186] Piccaglia R., Marotti M., Grandi S. (1998). Lutein and lutein ester content in different types of *Tagetes patula* and *T. erecta*. Ind. Crops. Prod..

[187] Del Campo J.A., Rodriguez H., Moreno J., Vargas M.A., Rivas J., Guerrero M.G. (2001). Lutein production by *Muriellopsis* sp. in an outdoor tubular photobioreactor. J. Biotechnol..

[188] Shi X.M., Zhang Z.H., Chen F. (2000). Heterotrophic production of biomass and lutein by *Chlorella protothecoides* on various nitrogen sources. Enzyme Microb. Technol..

[189] Del Campo J.A., Garcia-Gonzalez M., Guerrero M.G. (2007). Outdoor cultivation of microalgae for carotenoid production: Current state and perspectives. Appl. Microbiol. Biotechnol..

[190] Sánchez J.F., Fernández J.M., Acién F.G., Rueda A., Pérez-Parra J., Molina E. (2008). Influence of culture conditions on the productivity and lutein content of the new strain *Scenedesmus almeriensis*. Proc. Biochem..

[191] Sánchez J.F., Fernández-Sevilla J.M., Acién F.G., Cerón M.C., Pérez-Parra J., Molina-Grima E. (2008). Biomass and lutein productivity of *Scenedesmus almeriensis*: Influence of irradiance, dilution rate and temperature. Appl. Microbiol. Biotechnol..

[192] Kanazawa K., Ozaki Y., Hashimoto T., Das S.K., Matsushita S., Hirano M., Okada T., Komoto A., Mori N., Nakatsuka M. (2008). Commercial-scale Preparation of Biofunctional Fucoxanthin from Waste Parts of Brown Sea Algae *Laminalia japonica*. Food Sci. Technol. Res..

[193] Kim M.S., Kang S.W., Kwon O.N., Chung D., Pan C.H. (2012). Fucoxanthin as a major carotenoid in *Isochrysis aff. galbana*: Characterization of extraction for commercial application. J. Korean Soc. Appl. Biol. Chem..

[194] Kim S.M., Jung Y.J., Kwon O.N., Cha K.H., Um B.H., Chung D., Pan C.H. (2012). A potential commercial source of fucoxanthin extracted from the microalga *Phaeodactylum tricornutum*. Appl. Biochem. Biotechnol..

[195] Xia S., Wang K., Wan L., Li A., Hu Q., Zhang C. (2013). Production, Characterization, and Antioxidant Activity of Fucoxanthin from the Marine Diatom *Odontella aurita*. Mar. Drugs.

[196] Hurst D., Børresen T., Almesjö L., De Raedemaecker F., Bergseth S. (2016). Marine Biotechnology Strategic Research and Innovation Roadmap: Insights to the Future Direction of European Marine Biotechnology.

